# *Halobacterium salinarum* and *Haloferax volcanii* Comparative Transcriptomics Reveals Conserved Transcriptional Processing Sites

**DOI:** 10.3390/genes12071018

**Published:** 2021-06-30

**Authors:** Amr Galal Abd El-Raheem Ibrahim, Ricardo Z. N. Vêncio, Alan P. R. Lorenzetti, Tie Koide

**Affiliations:** 1 Department of Computation and Mathematics, Faculdade de Filosofia, Ciências e Letras de Ribeirão Preto, Universidade de São Paulo, Ribeirão Preto 14040-900, Brazil; amrgalal@usp.br (A.G.A.E.-R.I.); rvencio@usp.br (R.Z.N.V.); 2 Department of Biochemistry and Immunology, Ribeirão Preto Medical School, Universidade de São Paulo, Ribeirão Preto 14040-900, Brazil; alorenzetti@usp.br

**Keywords:** transcript processing sites, TPS, RNA processing sites, dRNA-seq, *Halobacterium salinarum* NRC-1, *Haloferax volcanii* DS2, comparative transcriptomics, post-transcriptional regulation, gene expression

## Abstract

Post-transcriptional processing of messenger RNA is an important regulatory strategy that allows relatively fast responses to changes in environmental conditions. In halophile systems biology, the protein perspective of this problem (i.e., ribonucleases which implement the cleavages) is generally more studied than the RNA perspective (i.e., processing sites). In the present in silico work, we mapped genome-wide transcriptional processing sites (TPS) in two halophilic model organisms, *Halobacterium* *salinarum* NRC-1 and *Haloferax* *volcanii* DS2. TPS were established by reanalysis of publicly available differential RNA-seq (dRNA-seq) data, searching for non-primary (monophosphorylated RNAs) enrichment. We found 2093 TPS in 43% of *H. salinarum* genes and 3515 TPS in 49% of *H. volcanii* chromosomal genes. Of the 244 conserved TPS sites found, the majority were located around start and stop codons of orthologous genes. Specific genes are highlighted when discussing antisense, ribosome and insertion sequence associated TPS. Examples include the cell division gene *ftsZ2,* whose differential processing signal along growth was detected and correlated with post-transcriptional regulation, and biogenesis of sense overlapping transcripts associated with IS*200*/IS*605*. We hereby present the comparative, transcriptomics-based processing site maps with a companion browsing interface.

## 1. Introduction

Post-transcriptional processing of mRNAs is an important regulatory mechanism of gene expression [1,2]. The phenomenon can be seen from two complementary viewpoints: the protein and RNA perspectives. For halophilic archaea, RNA-binding proteins, such as chaperones, and exo- and endoribonucleases, were characterized and the genetic perturbation was assessed at the systems-level [3,4], giving a general view from the protein perspective. On the other hand, although decay and turnover rates have been measured at the systems-level [5], there are no comprehensive genome-wide maps of processing sites comparable to that available for the methanogenic psychrophilic archaeon *Methanolobus psychrophilus* [6] or the hyperthermophile *Pyrococcus furiosus* [7], which would address the problem from the RNA perspective. Halophile research would benefit from the availability of such processing site maps for its two main model organisms, *Halobacterium salinarum* and *Haloferax volcanii*.

*Halobacterium salinarum* is a photosynthesizing archaeon that does not rely on either chlorophyll or bacteriochlorophyll [8]. It shows no turgor pressure and uses the “salt-in” strategy to achieve osmotic balance [9]. *H. salinarum* is an obligatory halophile that grows optimally at 4.3 mol/L NaCl concentrations and gets lysed at low salt concentrations. Its intracellular concentrations of K^+^ and Na^+^ are measured as ≈4 mol/L and ≈1.4 mol/L, respectively, with Cl^-^ being just 10% higher than the growth medium [10]. Classical and modern biological and biotechnological achievements were undertaken using *H. salinarum* as a model, ranging from the structural elucidation of bacteriorhodopsin [11] to vaccine improvement [12]. These achievements leveraged the proposal of an environmental/gene regulatory influence network that can predict transcriptional changes to new environmental or genetic perturbations [13,14] and consolidated *H. salinarum* as a model organism for gene regulation among archaea. *Haloferax volcanii*, on the other hand, grows optimally at 1.7 to 2.5 mol/L NaCl, a relatively moderate salt requirement in the halophile context [15]. *H. volcanii* is incapable of phototrophic growth or floating using a gas vesicle system, but forms biofilms and is more genetically stable due to less numerous insertion sequences [16]. It is able to produce most of its amino acids and degrade a set of sugars that *H. salinarum* cannot metabolize [15,16]. Overall, *H. volcanii* is a valuable model organism for its practical aspects [15], even participating in the early moments of the ongoing CRISPR revolution [17]. The amount of transcriptome data available for *H. salinarum* and *H. volcanii* is unparalleled in the third domain of life (≈53% of experiments and ≈24% of RNA entries for archaea at NCBI’s Gene Expression Omnibus and Sequence Read Archive databases, respectively, in January 2021).

Classically, a transcriptomics experiment is designed to understand the dynamic behavior of messenger transcripts as a proxy for protein-level changes. However, high-resolution hybridization (tiling microarrays) and sequencing-based (RNA-seq) transcriptome measuring technologies were able to reveal new genomic features that are difficult to be identified only by computational analysis of DNA sequences [18,19], expanding the scope of traditional transcriptomics to beyond differential expression. With an exponential decrease in costs, sequencing platforms were used in combination with several kinds of sample preparation strategies to explore different aspects of the transcriptome, creating a family of specialized RNA-seq protocols [20,21]. One such protocol is the differential RNA sequencing (dRNA-seq) method. The dRNA-seq method works by comparing differences between TEX (Terminator 5′ phosphate dependent exonuclease) treated samples against control samples in search of differential enrichment of 5′ triphosphorylated ends, thus unveiling primary transcripts. However, often neglected information that could be provided by the same protocol is the set of enriched 5′ monophosphorylated transcripts, which thus, unveils processed transcripts. Specific protocols for the efficient detection of RNA processing sites were developed (RiboMeth-seq [7], 5′P-seq [6] or pRNA-seq [22]), but legacy dRNA-seq datasets, originally deployed to map transcription start sites (TSS), can be readily reanalyzed. Instead of comparing an exonuclease-treated (TEX+) enrichment signal against a non-treated (TEX−) control (thus, TEX+ > TEX−), a search for TEX−mediated depletion signals (thus, TEX− > TEX+) were carried out, identifying transcriptional processing sites (TPS) [23].

In this work, we mapped TPS along the genomes of two halophilic model organisms, *Halobacterium salinarum* NRC-1 and *Haloferax volcanii* DS2, using previously acquired dRNA-seq data [24,25] and performed comparative transcriptomics to assess conservation. Finally, the processing site map just built was used to highlight specific cases in which post-transcriptional regulation seems to be relevant.

## 2. Materials and Methods

### 2.1. dRNA-seq Raw Data Alignment Reanalysis

Raw dRNA-seq data from *H. salinarum* NRC-1 [24] and *H. volcanii* DS2 [25] were reanalyzed with our in-house pipeline (“Caloi-seq”, https://github.com/alanlorenzetti/frtc/ (accessed on 27 May 2021)) as a byproduct of our group’s previous work on internal TSS (iTSS) and intraRNAs [24]. Although not fully explored in that work, which focused on iTSS, the pipeline created alignment files that are useful for the current work and that are considered the input data for our current reanalysis. Briefly, the read quality was inspected using FastQC v0.11.7; paired-end libraries were processed using Trimmomatic v0.36; reads that passed the filters as R1-R2 pairs were aligned to *H. salinarum* NRC-1 and *H. volcanii* DS2 reference genomes (NCBI’s Assembly accessions ASM680v1 and ASM2568v1) using HISAT2 v2.1.0 in paired-end mode. Orphan reads R1 and R2 that passed the trimming process but not as pairs were aligned using single-end mode. The resulting alignment files were used as input for MMR to treat multi-mapped reads and the output was submitted to an additional filtering step to keep only the aligned R1 reads. Furthermore, for visualization purposes, we processed MMR output files using deepTools v2.5.3 [26] to compute the genome-wide read coverage. The raw input files, under the accession numbers SRP137801 and SRP076059, were obtained from NCBI’s SRA database.

### 2.2. Transcript Processing Site (TPS) Mapping

We used TSSAR v1.0.1 [27] to identify TPS positions, deliberately changing the tool’s TEX+ input for the TEX− R1 alignment file and vice versa in order to highlight statistically significant 5′ monophosphorylated depletion signals (TEX− > TEX+). Therefore, TSSAR’s “TSS” outputs are actually TPS. TSSAR parameters were *p*-value (*p*) of *p* < 10^−9^ with a minimum of 10 reads and a distance of “TSS” grouping of at least 5 nt.

### 2.3. TPS Conservation

TPS were defined as “conserved” if the normalized position *D* in orthologous gene pairs (from the 1554 available at OrtholugeDB [28]) was less than two units apart (as introduced in [29]): |*D*_Hsa_ − *D*_Hvo_| < 3. The normalized position is the genomic coordinate *x*, where the TPS was found, divided by the CDS length: *D* = 100 |*x*_CDSstart_ − *x*_TPS_|/|*x*_CDSstart_ − *x*_CDSend_|; therefore, it is standardized within the interval zero and 100 if TPS is exactly at the start codon or at the last CDS nucleotide, respectively. Moreover, *D* < 0 and *D* > 100 were allowed to represent out-of-bound TPS at the 5′ and 3′ of a CDS, respectively.

### 2.4. Secondary Structure Prediction

Secondary structure predictions were carried out, using RNAfold v2.4.8 at the RNAfold web server [30] using default parameters, except for energy parameters, where “Turner model, 1999” was selected. For a few arbitrarily selected sequences, additional confirmatory predictions were carried out using the RNAbow, AllPairsMFE method with default parameters [31]. In order to highlight only high-probability base pairings in specific cases, we used the RNAstructure, ProbablePair method, with a > 0.99 probability cutoff [32]. RNA multiple alignment was carried out using LocARNA-P [33] with the “Turner model, 1999” energy parameters.

### 2.5. Gene Set Enrichment Analysis

Gene functional classification and Gene Ontology enrichment analysis were performed using the PANTHER system [34] at http://pantherdb.org/ (accessed on 28 March 2019) The default cutoff of the False Discovery Rate (FDR) of <5% was used in the Binomial test in the PANTHER Overrepresentation Test suite (Released 20181113). In order to avoid biases toward the orthologous genes’ functional classes, only categories/terms enriched that are not also enriched in an orthologous vs. whole genome comparison were considered.

### 2.6. Ribo-seq Data Analysis

*H. salinarum* NRC-1 ribosomal profiling (Ribo-seq) data were reanalyzed using the same pipeline used for dRNA-seq without special treatment, which is usually deployed when dealing with translation initiation site identification. In order to generate the genome browser visualization, all time point samples and their replicates were combined by summing their read coverage. The data are publicly available under NCBI’s SRA accession SRP119792 [35].

### 2.7. Differential Expression and Qualitative Analysis

The expression profiles along the genome coordinates were considered mostly qualitatively and the signal profiles were evaluated visually using the Gaggle Genome Browser [36] tool, with no special attention to numeric *y*-axis scales or normalization, except when comparisons between libraries or experiments were made. All expression profiles were examined in log_2_ or absolute scales with appropriate identification. Traditional *H. salinarum* RNA-seq data, a complementary dataset published along with Ribo-seq data (SRP119792), were used in specifically marked cases. *Natrinema* sp. J7-2 differential expression analysis was carried out after signal normalization to ensure comparability between three experiments. The sum of coverage in all genomic positions was used as the normalization factor, so the signal integral was set to 1, re-scaled to “per million” for convenience and shown without log_2_ scale. The *H. salinarum* differential expression analysis used log_2_ and a simple normalization factor that rescaled the least sequenced libraries to match the most sequenced library.

## 3. Results

### 3.1. Transcript Processing Site (TPS) Mapping

We identified transcript processing sites (TPS) in *H. salinarum* NRC-1 and *H. volcanii* DS2 by reanalyzing public dRNA-seq. We searched for TEX+ induced depletion signals using the TSSAR v1.0.1 stand-alone tool [27]. High number of sequencing reads starting at a specific given position in TEX− libraries that were depleted in TEX+ libraries are signatures of 5′ monophosphorylated molecules and, therefore, the outcome of a cleavage event of a longer precursor. In order to properly explore the generated map, interactive stand-alone Gaggle Genome Browser [36] interfaces were created for both organisms. The interfaces were also used as a source for some of the figures and supplemental figures (Supplemental Files S1 to S4; available at http://labpib.fmrp.usp.br/~amr/tps/ (accessed on 27 May 2021)).

This approach identified 2098 processing sites in 1183 coding sequences (CDS) in *H. salinarum* (Table S1). This number represents 43% of all 2782 CDS considered (annotation from [24]). Most genes showed a single TPS (59%) and almost all genes presented, at most, three TPS (91%). Only two genes had more than 10 TPS: a halolysin (VNG_RS10060 *locus*) and an integrase (VNG_RS00870). The region with the highest TPS density (8 events in 200 nt, 0.04 nt-1) is associated with an RNase H domain containing exoribonuclease (VNG_RS04745, Figure S1a).

**Figure 1:**
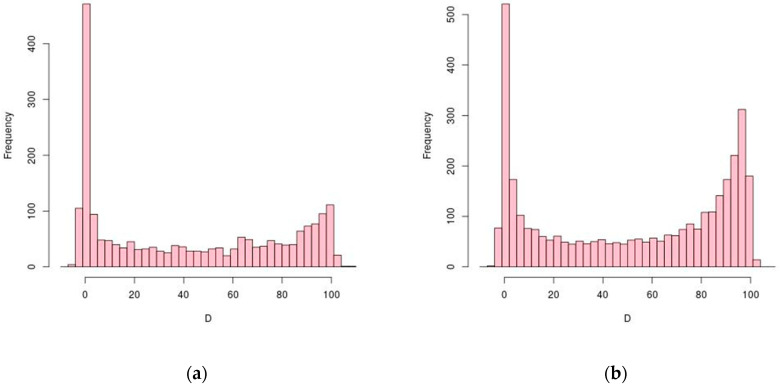
Distribution of CDS length-normalized positions (*D*) of TPS (transcriptional processing sites). Processing sites at start and stop codons are *D* = 0 and *D* = 100, respectively. Histograms have size 3 bins. (**a**) *H. salinarum* NRC-1; (**b**) *H. volcanii* DS2.

In order to learn about the spatial distribution of TPS along genes, each TPS coordinate was normalized considering its cognate CDS length, creating a relative position coordinate system. Values 0 and 100 indicate TPS at the start and stop codons, respectively, and values outside this range are allowed to account for putative 5′ and 3′ UTR processing. Figure 1a shows the overall distribution of TPS positions along *H. salinarum*’s CDS in which most of the TPS are located near the start codons.

**Figure 2:**
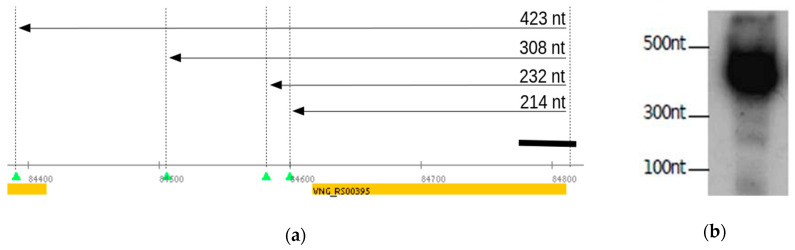
Experimental validation of predicted TPS using gene *cspA1* (VNG_RS00395 *locus*). (**a**) The consecutive TPS predict mRNAs of various sizes (arrows) from TSS to TPS_8361_1, TPS_8363_1, TPS_8365_1 and TPS_8366_1. Northern blot probe location is marked by the black rectangle. (**b**) Northern blot adapted from Figure 3 in [37].

The reanalysis of *H. volcanii* data yielded 3515 TPS distributed in 1408 CDS (Table S2) out of 2828 chromosomal CDS considered (49%; RefSeq annotation for ASM2568v1). Genes with a single TPS were the most common (45%) and cases showing at most three TPS were the majority (79%). Figure 2b shows the distribution of TPS normalized positions along *H. volcanii*’s CDS, showing a preferential location near the start codon as in *H. salinarum*, and a higher concentration near the stop codons. The region with the highest TPS density (11 in 200 nt, 0.055 nt^−1^) is surrounding the chaperone *cspA4* gene’s stop codon (HVO_RS14265 *locus*, Figure S1b).

Here, we focused only on annotated CDS and adopted a relatively stringent statistical significance cutoff, *p* < 10^−9^, an inclusion criterion that, if relaxed, could result in more TPS candidates (Tables S3 and S4). Moreover, tRNAs or rRNAs, which are not the focus of this work, are well-known cases of intense processing and, as expected, harbor plenty of TPS signals (see Supplemental Files S1 and S2 for exploration in the genomic context).

Aiming the validation of our approach, we reused the previously published Northern blot results from our group in which some of the detected bands can now be explained by the TPS analysis. Back then, the *cspA1* gene, which encodes for a highly conserved chaperone protein (VNG_RS00395 *locus*), was probed to demonstrate the ubiquitous presence of TSS associated RNAs (TSSaRNA) in all three domains of life [37]. However, it was left unexplained in [37]: (i) the fainter bands longer than the TSSaRNA and (ii) the strongest signal between 300 and 500 nt, much longer than the 195 bp CDS. Transcripts starting at the genuine TSS (5 nt 5′UTR) and ending at TPS would result in truncated molecules of 423, 308, 232 and 214 nt in length, respectively (Figure 2a and Figure S2a). This prediction is consistent with the experimental observation of bands between the 500, 300 and 100 nt molecular markers (Figure 2b, adapted from Figure 3 in [37]).

Similarly, processing sites provide reasonable explanations for the previously published, seemingly odd Northern blot results: the conserved arginine deiminase pathway *arcRACB* gene cluster was interrogated by different probes revealing diverse isoforms, two of which unusually started inside *arcA,* forming *arcA’CB* and *arcA’’CB* transcripts (Figure 4C in [38]). These 25-year-old results are remarkably consistent with sites TPS_20932_1 and TPS_193446_1 (Figure S2b).

Cases in *H. volcanii* can also be mined from the literature. The published Northern blots showing fragmented expression patterns of the polycistronic *tsg* operon, which encodes an ABC-type sugar transport system, validated our TPS finding approach: the dRNA-seq reanalysis showed processing break-points that would result in 7.1, 5.2, 3.8, 2.9, 1.5, and 1.9 knt transcripts that are consistent with experimental bands and the original authors’ hypotheses of the partial termination and processing of a large polycistronic primary transcript, resulting in several transcripts observed by Northern blot (from operon’s TSS to TPS_086838, TPS_086886, TPS_086946, TPS_086991 and TPS_087101 at the 5′-end part; from TPS_086892 to TPS_086838 at the 3′-end part; matching with Figure 6b,d in [39], among other combinations).

Similar observations can be made even between different organisms. The published Northern blot results for gas vesicle system genes in the moderately halophilic microorganism *Haloferax mediterranei* showed different transcripts isoforms arising from the *gvpDEFGHIJKLM* operon [40], consistent with our *H. salinarum* TPS data (*H. volcanii* has no gas vesicle system). The cleavage breakpoint in *H. mediterranei*’s transcript that generated the *gvpDE*’ (2 kb) processed transcript is equivalent to *H. salinarum*’s TPS_19964_1 (VNG_RS10615 *locus*), which in turn predicts a 2087 nt transcript in *H. salinarum* (Figure S2c). Analogously, the two *H. mediterranei* 0.45 and 1.3 kb *gvpD*’ transcripts are consistent with TPS_18331_1 and TPS_19982_1, respectively. Additional validation is provided by TPS found within the main gas vesicle system gene *gvpA1* (VNG_RS10625 *locus*): it is located at the basis of a stem–loop structure, experimentally implicated in the mRNA stability [40]. This stem–loop is 64 nt upstream to the stop codon in *H. mediterranei* and the TPS is 61 nt upstream to the stop codon in *H. salinarum* (Figure S2d).

Taking together, these results validate our dRNA-seq re-purposing to find transcript-processing sites (TPS).

### 3.2. TPS Conservation

*H. salinarum* and *H. volcanii* are both halophiles but they have distinct biological properties, including requirement/tolerance in environmental salt concentrations. They belong to the same phylum and class (Halobacteria) but to different orders (Halobacteriales and Haloferacales). Therefore, we assume that the TPS present in both organisms are probably relevant.

We defined TPS as “conserved” if the relative normalized position *D* in which they are found in orthologous gene pairs are approximately the same (i.e., |*D*_Hsa_ − *D*_Hvo_| < 3, in a 0 to 100 scale). This criterion yielded 244 TPS (Table S5) in 178 orthologous gene pairs out of 1554 available at OrtholugeDB [28].

Enrichment analysis [34] using all genes as background showed that Gene Ontology (GO) terms are overrepresented only in the 178 orthologous genes bearing conserved TPS but not in the whole orthologous gene set (Table S6). Among these, we highlighted the most enriched “biological processes”, “molecular functions” and “cellular components” GO branches, respectively; all with approximately 10-fold enrichment with a false discovery rate (FDR), controlled at less than 5%: iron–sulfur cluster assembly (GO:0016226); ATPase activity, coupled to transmembrane movement of ions, rotational mechanism (GO:0044769); and proton-transporting two-sector ATPase complex (GO:0016469).

**Figure 3:**
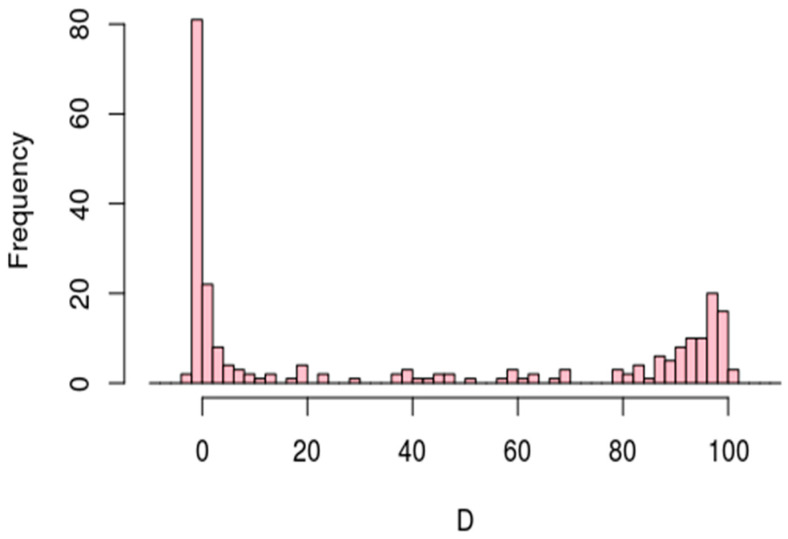
Distribution of CDS length-normalized positions (*D*) of TPS conserved between *H. salinarum* NRC-1 and *H. volcanii* DS2. Processing sites at start and stop codons are *D* = 0 and *D* = 100, respectively.

Conserved TPS are concentrated upstream of start codons and around stop codons (Figure 3). The histogram of *D* for conserved TPS has similar characteristics of general TPS (Figure 1).

### 3.3. H. salinarum Specific Internal TPS

Since *H. salinarum* and *H. volcanii* are organisms that show important lifestyle differences, we then evaluated the orthologous genes with internal TPS present in one but not in the other, with special attention to salt homeostasis related genes, which may indicate differential post-transcriptional regulatory strategies.

Out of the 1554 orthologous gene pairs, 74 *H. salinarum* genes had internal TPS (25 < *D* < 75) with no counterpart in the *H. volcanii* equivalent region (Table S7). Reciprocally, 165 *H. volcanii* genes had internal TPS with no *H. salinarum* counterparts (Table S8). Gene set enrichment analysis showed no significant output among the *H. salinarum* specific internal TPS and just one molecular function in the *H. volcanii* case: ATPase activity, coupled to transmembrane movement of ions, rotational mechanism (GO:0044769, 12-fold enrichment, FDR < 2%).

From the *H. salinarum* specific internal TPS set, we highlight the *kef1* gene, which encodes a sodium transporter protein (TPS_13995_1, VNG_RS07995 *locus, D* = 73). We did not find an equivalent TPS in the moderate halophile *H. volcanii*, but there is evidence for an internal transcript in two other non-moderate halophilic archaea: *Natrinema* sp. J7-2 and *Haloquadratum walsbyi*. *H. walsbyi*, a hyperhalophilic organism isolated from a brine pool in Egypt [41], was probed under different illumination conditions by regular RNA-seq [42]. This experiment revealed a coverage signal peak around an equivalent position inside *kef1* (HQ_RS10745 *locus*, *D* = 71) (Figure S3b). *Natrinema* sp. J7-2 (formerly known as *H. salinarum* J7) is an extremophile isolated from a Chinese salt mine that requires, at least, a 10% and optimally 25% salt concentration to grow. Its transcriptome was studied by regular RNA-seq in three salinity conditions: low (15% NaCl, 2.6M), optimal (25% NaCl, 4.3M) and high (30% NaCl, 5.1M). Reanalysis of these data showed not only signals of an equivalent TPS inside *Natrinema* sp. J7-2′s *kef1* gene (NJ7G_RS00730 *locus*, *D* = 74) but also that the cleavage output varies according to the environmental salt concentration (Figure S3c). We observed the same trend in our replication of this experiment in *H. salinarum* NRC-1: TPS_13995_1 increases > 4-fold in low salt relative to optimal growth condition (Onga et al., in prep.).

**Figure 4:**
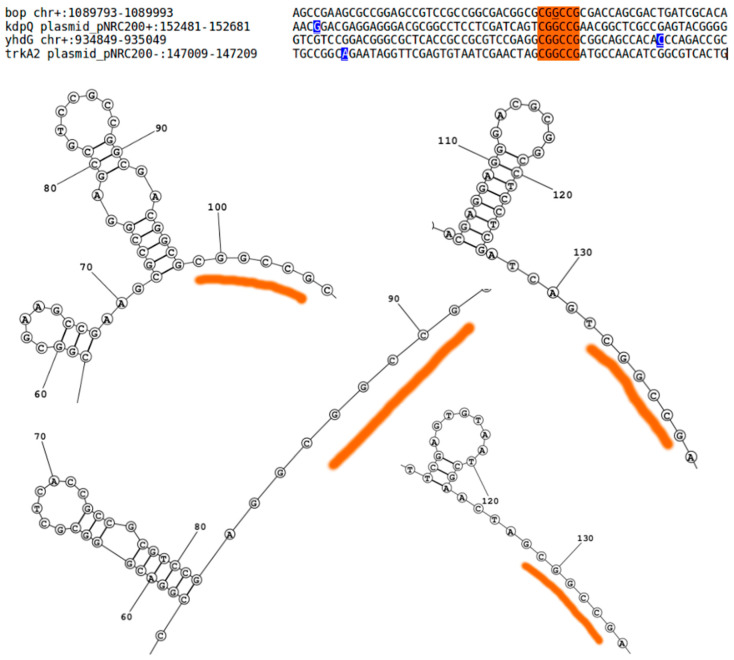
Putative signature found in dysregulated genes after VNG2099C RNase deletion using structure-aware RNA multiple alignment guided by TPS sites. Sequences from −100 to +100 around TPS (blue underlined bases) inside genes *bop*, *kdpQ*, *yhdG* and *trkA2* were used as alignment input. All genes harbor the CGGCCG motif downstream to a strong stem-loop (red highlights). The secondary structure predictions were filtered to report only base pairings with >0.99 probability (zoomed out version in Figure S4). Secondary structure predictions are, from top left to bottom right, for genes: *bop*, *kdpQ*, *yhdG* and *trkA2*.

Given the importance of RNA processing to the salinity adaptation of *H. salinarum* [4], we investigated the association between internal TPS and the endoribonuclease activity of a regulatory RNase, namely VNG2099C (VNG_RS08138 *locus*). It was shown that the archaeal RNase VNG2099C contributes to growth, the regulation of ion transport and is involved in environment-dependent physiologic transitions [4]. Four main genes highlighted in that study were relevantly dysregulated by VNG2099C deletion: *bop*, which encodes the light-driven proton-pump bacteriorhodopsin (VNG_RS05715 *locus*); *kdpQ*, encoding a positive auto-regulator for the Kdp potassium transport channel (VNG_RS11195); *trkA2*, encoding one regulatory subunit of a H^+^-K^+^ symporter (VNG_RS11170); and *yhdG*, encoding an ornithine-arginine antiporter (VNG_RS04855). We found TPS in *bop*, *kdpQ* and *trkA2.* Only *yhdG* did not have a TPS automatically identified by our dRNA-seq reanalysis since it is borderline statistically significant (TPS_3805_1, *p* = 2.8 10^−6^ > 10^−9^), although clearly present (Figure S4d). A sequence-based common feature among these genes was not reported before and our own extensive multiple alignment or Markov chain-based motif search could not detect putative cleavage sites either. However, conjecturing that the TPS found inside those genes are related to VNG2099C’s endonuclease activity, and performing structure-aware RNA multiple alignment [33] around these TPS sites, we were able to identify a signature: CGGCCG downstream of a strong stem–loop secondary structure (Figure 4).

Although it is not clear how the VNG2099C RNase mechanism generates these cuts, the sequence-structure putative signature paves the way for future research.

### 3.4. TPS Relationship with Gene Expression during Growth

**Figure 5:**
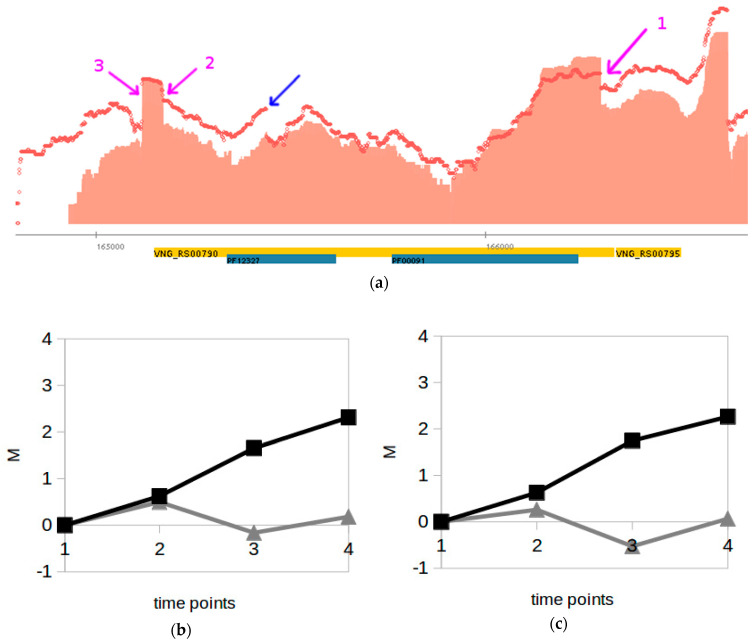
Example of differential processing during *H. salinarum* growth. (**a**) The conserved TPS (TPS_8657_1, arrow 1) is near the start codon inside the *ftsZ2* gene (VNG_RS00790 *locus*) which encodes a cell division protein. Pfam domain annotation (blue rectangles) and coding sequences are in reverse strand (orange rectangles), thus, 5′→3′ direction is right to left. Aligned reads coverage along genomic coordinates for TEX− libraries at exponential and stationary phases are shown in light red (solid) and red (dots), respectively (log_2_ counts normalized and arbitrarily jointly scaled). Magenta arrows 2 and 3 point to non-conserved TPS and blue arrow points to intraRNA. (**b**) *ftsZ2* gene log_2_ fold-change (M) between multi-modality measurements in different time points relative to the early exponential phase from [35] (time point 1: early exponential, 2: mid exponential, 3: late exponential, 4: stationary, squares: RNA-seq data, triangles: Ribo-seq data). (**c**) Same as (**b**) but for *cdrS* (VNG_RS00795 *locus*), encoding a *ftsZ2* regulator.

*H. salinarum* dRNA-seq data were acquired at different time points across a typical growth curve experiment (Figure S5a), allowing a time-dependent comparison of processing products abundance. Individual inspection of all 244 conserved TPS at the exponential phase (17 h, OD600 ≈ 0.3) versus stationary phase (37 h, OD600 ≈ 0.5) showed that the vast majority of the transcripts starting at these positions follow the same expression patterns of their cognate full-length genes (File S3). Four notable exceptions (Figure 5 and Figure S5) are genes: *ftsZ2*, which encodes a cell division protein (TPS_8657_1, VNG_RS00790 *locus*); *eEF1A*, encoding an elongation factor (TPS_16108_1, VNG_RS10385); a putative arsenic resistance operon repressor encoded at the VNG_RS03675 *locus* (TPS_2832_1); and *pcn*, encoding a DNA polymerase III subunit (TPS_14733_1, VNG_RS08800).

In halophilic archaea, the operon *cdrS-ftsZ2* is highly conserved and *cdrS* regulates *ftsZ2,* a tubulin homolog with a pivotal role in cell division [43]. A comparison among quantitative transcriptome (RNA-seq) and ribosome occupancy (Ribo-seq) data taken from the same *H. salinarum* growth time course [35] showed that this operon is probably subject to post-transcriptional regulation (Figure 5b,c). Although mRNA levels increase over time, as seen in the dRNA-seq dataset (Figure 5a), ribosome protected fragment levels indicated that they are not translated in the same proportion (Figure 5b). The TPS location inside *ftsZ2* near the 5′ end region, coinciding with a decrease in the RNA-seq signal in the stationary phase (Figure 5a), indicates that mRNA processing is involved in post-transcriptional regulation. The possibility of asRNA-mediated processing was investigated since in *E. coli,* the asRNA interaction with *ftsZ2* 5′-end regulates cell division [44]. The dRNA-seq data we reanalyzed here show clear signs of an asRNA presence in the vicinity of *ftsZ2* gene 5′-end in *H. volcanii* (HVO_RS07495) but not in *H. salinarum* (Files S1 and S2). Interestingly, there is an intraRNA [24] that increases its expression over time (Figure 5a, blue arrow) but the region flanked by two TPS, TPS_8628_1 and TPS_8625_1, remained constant (Figure 5a).

Although not the main focus here, there are differential processing cases in genes conserved between *H. salinarum* and bacteria, but not present in *H. volcanii*. An interesting example is *arcA* (VNG_RS11635), a gene involved in the arginine deiminase pathway, which allows fermentative arginine utilization by many bacteria and has been acquired by *H. salinarum* and not by *H. volcanii*. This gene was mentioned in a previous section to support the validation effort (Figure S2b). A previous study showed conditional independence of transcription inside the *arcRACB* cluster with clear transcription factor binding sites just upstream to all the genes [45]. Here, we noticed that along such “synthesis” signs, there are also “breaking” signs with TPS spread along the cluster, specially at 5′-ends inside or outside the coding regions. We speculate that the equilibrium between binding and processing would calibrate the appropriate *arcRACB* stoichiometry according to environmental conditions. In spite of the great increase in *arcA* transcript abundance along the growth curve, the signal pattern around the TPS is different (Figure S6a).

### 3.5. Antisense Transcript Processing Site (aTPS) Mapping

We identified antisense transcription processing sites (aTPS) in *H. salinarum* NRC-1 and *H. volcanii* DS2, expecting that a subset of them would be signatures of sense/antisense hybridization followed by cleavage events. A total of 265 and 629 aTPS were found in *H. salinarum* and *H. volcanii*, respectively (Table S9 and Table S10).

From a sample of 8 chromosomal asRNAs validated by Northern blot and conjectured to be processed products in *H. volcanii* [46], 6 are consistent with aTPS found here: HVO_2293, HVO_2990, HVO_1027, HVO_0263, HVO_0599, HVO_2928. This validation result, together with what we observed for regular TPS, is an indication that our approach is also reasonable for aTPS finding.

Comparative transcriptomics considering Escherichia coli str. K-12 substr. MG1655, for which sense/antisense interaction was established [47] indicates that aTPS can point to double-stranded RNA (dsRNA). An example of such a phenomenon is found within the stress-related CspA (CSD domain containing) family genes. All 4 of the H. volcanii chromosomal cspA genes presented at least one aTPS along with H. salinarum cspA1 (VNG_RS00395 locus). In E. coli, sense/antisense hybrids were experimentally observed for cspD, cspE, cspF, cspH, members of CspA family that are not directly involved in the cold shock response [47]. Similarly, in H. mediterranei, Northern blot data for arcA gene show a remarkable co-localization between experimentally detected long-processed product and an aTPS counterpart in H. salinarum (Figure S2b, TPS_19346_1) (this gene is absent in H. volcanii). Evidently, the presence of an aTPS is not a definitive hallmark of asRNA interaction, but rather is additional, circumstantial evidence.

**Figure 6:**
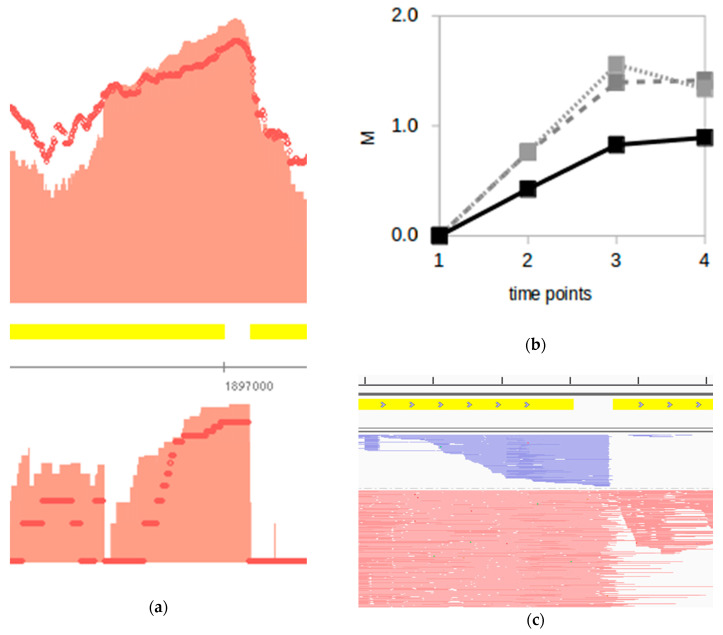
Example of sense/antisense processing during *H. salinarum* growth. (**a**) TPS (TPS_7176_1, top panel) and aTPS (TPS_15790_1, bottom panel) are located between *dppA2* and *dppB2* (VNG_RS09900 and VNG_RS09905 *loci*, 600 bp zoomed in out of a ~1.7 kbp gene), which encode members of a dipeptide ABC transporter system. Coding sequences are in forward strand (yellow rectangles) thus 5′→3′ direction is left to right, upper panel shows dRNA-seq data for forward strand and lower panel for reverse strand. (**b**) *dppA2, dppB2* and *dppC2* gene log_2_ fold-change (M) between transcriptome data in different time points relative to the early exponential phase from [35] (time point 1: early exponential, 2: mid-exponential, 3: late exponential, 4: stationary, filled black: *dppA2*, dashed grey: *dppB2* data, dotted light grey: *dppC2*). (**c**) Sample of aligned reads in the same window of (**a**) but not log scaled. Reads aligned to the reverse (antisense to *dppA2*) and forward strands are shown in blue and red, respectively (reads in red cropped vertically and horizontally for clarity since they are ~10-fold more abundant in this window). Tick marks are 100 bp apart.

A search for positional coincidence within a 5 nt window between TPS and aTPS as a potential signature of the sense/antisense interaction returned very few candidates: 18 and 48 pairs in H. salinarum and H. volcanii, respectively (Tables S11 and S12). Antisense transcripts are notoriously non-conserved and that is also the case between H. salinarum and H. volcanii [29]. Imposing that aTPS must be present in orthologous gene pairs, we found only 5 genes: dppA2, iscU, nosF1, atpI and VNG_RS08220 (loci pairs VNG_RS09900-HVO_RS07725, VNG_RS09660-HVO_RS05210, VNG_RS09315-HVO_RS19230, VNG_RS08320-HVO_RS06205, VNG_RS08220-HVO_RS06290, respectively). The case of dppA2 (alternatively, dppD), part of a dipeptide ABC transporter dppFABC operon, is particularly interesting since its constituents were shown to be conditionally regulated in H. salinarum (Figure 4D in [45]). Eventual decoupling between dppF and downstream dppABC was attributed to environment-dependent transcription factor binding combinations. However, checking regulatory and transcriptional data (raw intensity data from [45] and module hc2556 from [14]) we see that dppA2 can also be decoupled from dppBC. Our TPS analysis suggests that there is a dppABC cleavage and the aTPS presence allows us to speculate that this could be an asRNA-mediated process. Levels of dppA2, dppB2 and dppC2 rise along the growth curve in dRNA-seq and RNA-seq data (Figure 6) but available observable transcripts diminish around both TPS and aTPS regions (Figure 6a). Blunt sequenced antisense read edges match well the sense read 3′-ends (Figure 6c), consistent with a double-strand hybridization model.

### 3.6. TPS Associated with Ribosome Dynamics

From all 244 conserved TPS, at least 157 (64%) are inside or closely adjacent to *H. salinarum* Ribo-seq peaks (Table S13). Examples of such TPS/Ribo-seq coincidences are shown in Figure S7 for a few arbitrarily selected genes and the complete dataset can be explored using the File S4.

Interestingly, *pan1* (VNG_RS01995), which encodes the PAN-A proteasome-activating nucleotidase, has two alternative transcripts translated in two protein isoforms in *H. salinarum* [48] that correspond to the TPS found. The difference between both transcripts (Figure S7a) roughly matches the Ribo-seq peak and is consistent with conserved TPS locations in both *H. salinarum* (TPS_9619_1) and *H. volcanii* (TPS_058741, HVO_RS08770) considering the nominal positional uncertainty. Previous searches for ribosome associated ncRNAs (rancRNAs) in *H. volcanii* [49] led to the discovery of 856 *loci* internal to annotated CDS from which 56 (7%) coincide with the TPS hereby presented (Table S14). One of those rancRNAs is located between *pan1* transcript isoforms and we speculate that it may be involved in the choice for protein isoforms in both organisms.

Since Ribo-seq profiles along genomic coordinates can be reflexes of prolonged pauses or stalled ribosomes during the translation process [50], we hypothesize that these TPS found in the vicinity of Ribo-seq peaks may be related to “No-Go” decay [51].

### 3.7. TPS Identify ncRNAs Derived from Insertion Sequence Transcripts

Originally described in the extremophile model archaeon *H. salinarum* NRC-1, sot0042 RNA family (Rfam accession RF02656) is now annotated in 54 other species (144 sequences), mostly Halobacteria. These RNAs are transcribed from within insertion sequences (IS) in the same strand as their protein-coding genes. The previous effort to establish the sot0042 RNA family was not able to elucidate its biogenesis [52]. Given that Northern blot experiments detected VNG_sot0042 but not the cognate protein coding *tnpB* (VNG_RS00160 *locus*) full-length transcripts, the processing scenario was further investigated here.

**Figure 7:**
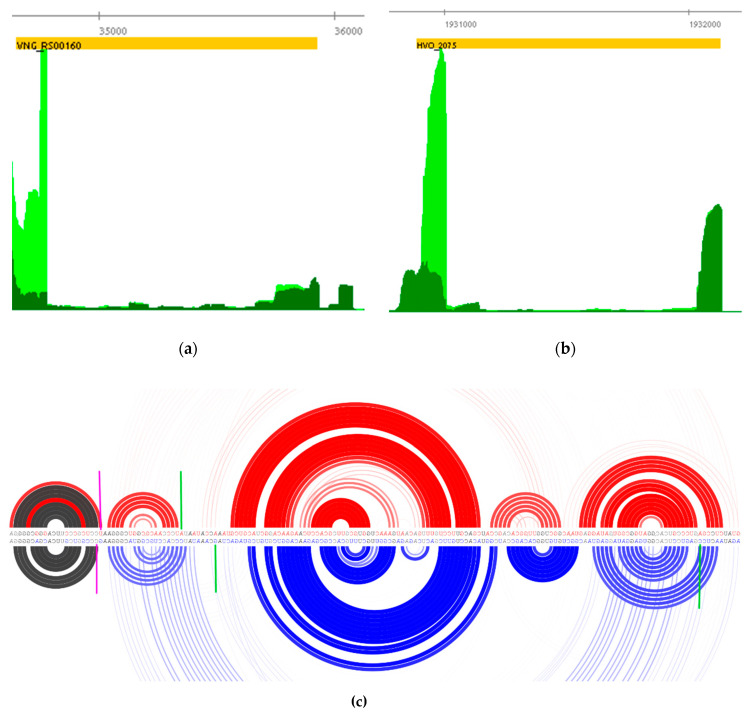
Gene *tnpB* transcript processing site signal (TEX− > TEX+) in (**a**) *H. salinarum* and (**b**) *H. volcanii* dRNA-seq datasets. Coding sequences are in reverse strand (orange rectangle), thus 5′→3′ direction is right to left. Aligned reads coverage along genomic coordinates for TEX+ and TEX− are shown in dark green and light green, respectively (normalized counts arbitrarily scaled). Statistical analysis showed the same results for the sot0042 family (RF02656) member within the VNG_RS00160 *locus* (TPS_8065_1) and HVO_2075 *locus* (TPS_077749). (**c**) Predicted rainbow diagram of sot0042 family representatives in *H. salinarum* (top, red) and *H. volcanii* (bottom, blue), 5′→3′ direction is right to left. Arc thickness is proportional to the thermal average probabilities of base pairs. Magenta markers point to hairpin induced false positive TSS. Green markers indicate transcript processing sites (TPS) inferred by statistically significant signal suppression after TEX treatment (right to left): TPS_077749, TPS_077728, TPS_8058_1.

A clear TEX+ < TEX− depletion signal (TPS_8065_1) is detected coinciding with the VNG_sot0042 start site, supporting the processing biogenesis hypothesis (Figure 7a). This observation is not restricted to the sot0042 RNA family but rather applies to almost all other sense overlapping RNAs (sotRNAs) previously found. Of all 10 sotRNAs previously identified near IS 3′ ends in *H. salinarum* [52], seven show clear TEX+ depletion as evidence of being processing products of their cognate IS transcripts: VNG_sot0026 (TPS_7956_1), VNG_sot0042 (TPS_8065_1), VNG_sot0044 (TPS_263_1), VNG_sot0286 (TPS_1226_1), VNG_sot6181 (TPS_20588_1), VNG_sot6221 (TPS_20696_1) and VNG_sot6361 (TPS_19531_1). From the remaining three out of 10 cases, VNG_sot0013 and VNG_sot2652 showed TEX+ depletion evidence (Figure S8) but were not automatically identified by our statistical pipeline. Generally, the *loci* coordinates inferred originally using sRNA-seq data [37] agreed with the current dRNA-seq based TPS analysis within a <5 nt margin. Analysis of public *H. volcanii* dRNA-seq data showed the same results (Figure 7b) for the sot0042 family member harbored within the HVO_2075 *locus* (TPS_077749).

Our observations are not limited to Haloarchaea but rather can also be seen in Thermococci and even in Bacteria. Our reanalysis of *Thermococcus kodakaraensis* KOD1 dRNA-seq data (PRJNA242777) shows strong evidence for sotRNA processing biogenesis for two out of all six IS*605*-related annotated transposases [52] (Figure S9). Similarly, reanalysis of several bacterial dRNA-seq data shows TPS inside the IS *locus* near the characteristic transposase DNA-binding protein domain OrfB_Zn_ribbon (PFAM database accession: PF07282). Considering over 950 bacterial species currently annotated as presenting PF07282 as their last C-terminal protein domain, at least five also had their primary transcriptome investigated by dRNA-seq: *Escherichia coli* K-12 (PRJNA238884), *Helicobacter pylori* 26,695 (PRJNA343039), *Streptomyces coelicolor* M145 (PRJNA285265), *Synechocystis* sp. PCC 6803 substr. GT-I (PRJNA224696) and *Mycobacterium tuberculosis* H37Rv (PRJEB1807). All of these five bacteria showed a clear TPS signal approximately at the same region near the PF07282 3′-end boundary (Figure S10), a feature also shown in our own data for the extremophile archaeon *H. salinarum*.

## 4. Discussion

The present bioinformatics study is embedded in the spirit of maximum data reuse. The identification of transcript processing sites (TPS) in *H. salinarum* NRC-1 and *H. volcanii* DS2 was achieved by reanalyzing public dRNA-seq data. Usually, dRNA-seq is performed to point to TEX+ > TEX− signals. However, using the same underlying statistical model, it is possible to additionally search for TEX− > TEX+ signals, yielding processing sites. Therefore, we used a well-established tool, TSSAR, forcefully inverting the labels of treated and untreated libraries to map putative TPS sites using the same published datasets. We validated this approach using published Northern blot experiments (Figure 2 and Figure S2) originally performed to support other scientific questions. The observed hybridization bands match well the putative transcripts with breakpoints at the TPS now identified.

TPS maps were made available through interactive files for the Gaggle Genome Browser (GGB), which renders navigation-enabled versions of Figure 2a, Figure 5a, Figure 6a and Figure 7 and so on. The GGB platform is easy to use, and further instructions are available at the companion website http://labpib.fmrp.usp.br/~amr/tps/ (accessed on 27 May 2021) along with dRNA-seq, Ribo-seq and the differential expression publicly available data used in this work in GGB format (Files S1 to S4). This is the main result of our work: a resource that can pave the way for diverse downstream research initiatives.

The majority of TPS are concentrated around 5′ ends. Both halophiles have a relatively high rate of leaderless transcripts (≈70%) [24,25], which is consistent with the observed low frequency of *D* < 0 cases. A second population is located around 3′ ends, especially in *H. volcanii*. Small- and large-scale studies on archaeal 3′ UTR indicated the regulatory relevance of such regions [53,54]; however, the majority of TPS found at 3′ ends are contained within CDS and not at UTR (*D* < 100). In bacteria, 3′ ends can also work as a reservoir of regulatory sRNAs [55]. Many of the *D* > 100 cases are located between consecutive genes in operons. This is consistent with what was found in *E. coli* using Term-seq data [56], where mRNA processing of genes within an operon creates differential transcript abundances for equally transcribed operon members. Differential transcription initiation of operon members was reported before in *H. salinarum* [45] but differential decay has not been observed [5]. However, an example of an intra-operonic differential processing case was shown in (dppFABC operon, Figure 6). RNA half-life measurements using massive sequencing platforms in multiple environmental conditions may resolve this issue. Alternatively, and as a possible technical limitation, it is possible that such 5′-end enrichment of TPS would be simply a reflection of sequencing selection bias.

Our approach detects transcripts or segments of transcripts that were cleaved from longer precursors, but it cannot elucidate the mechanism or biogenesis of such a molecular break. It is impossible to know whether detected transcripts are a result of stalled 5′→3′ exoribonuclease digestion or endoribonuclease cleavage since the final observable is the same. Exoribonucleases acting in the 3′→5′ direction are not expected to generate an enrichment of reads all starting at the same genomic position. The internal TPS, on the other hand, are expected to be results of endonuclease activity.

We carried out extensive unfruitful searches for sequence motifs that could unify all internal TPS. No specific endoribonucleolytic cleavage signal that initiates mRNA decay is known in spite of endoribonuclease-high conservation in archaea [1]. Therefore, it is reasonable to assume that more than one RNase and more than one mechanism are operational at the same time. Our approach is not able to discriminate each case. We hypothesize that much of the difficulty in identifying sequence targets for endonucleolytic RNases may be explained by the possibility that no specific sequence motif is recognized, but rather structural motifs. However, TPS could aid the localization of putative cleavage motifs if integrated with additional data: we managed to find a putative RNase cleavage signature related to physiologically relevant regulatory action, using published RNase deletion information (Figure 4) [4]. The principled sub-selection of sequences surrounding the TPS mapped in this work may be the starting point to find missing signatures.

We hypothesize that TPS located approximately at the same relative positions in orthologous genes between *H. salinarum* and *H. volcanii* could be functionally relevant or a product of a conserved post-transcriptional regulation mechanism. The distribution of conserved TPS locations (Figure 4) is similar to overall sets from both organisms (Figure 2).

Iron–sulfur cluster assembly, a virtually omnipresent process due to its importance in providing protein co-factors, was found to present TPS more often than other biological processes. In bacteria, the iron–sulfur assembly protein concentrations are regulated post-transcriptionally [57]. It is reasonable to hypothesize that the TPS found overrepresented in iron–sulfur cluster assembly-related genes are evidence of relevant post-transcriptional regulation. Relevant to their lifestyle, active transmembrane ion transport was also found to resort to post-transcriptional regulation more than other molecular activities, even when taking into account the bias over orthologous genes between *H. salinarum* and *H. volcanii*. The same molecular activity was found to be overrepresented when considering *H. volcanii-*specific TPS, which may indicate that active ion transport by a rotational mechanism is tightly controlled in this organism relative to *H. salinarum*.

One of the main differences between *H. salinarum* NRC-1 and *H. volcanii* DS2 is the gas vesicle system, present in the former but not in the latter. This system, composed by at least two structural proteins (GvpA and GvpC) and several regulatory proteins, allows some halophilic organisms to float and search for optimal conditions along the water column [40,58]. *H. volcanii* is often used as a model to study gas vesicle system genes through transformation and heterologous expression [15]. We found some TPS inside the main gas vesicle system genes and conjecture that the post-transcriptional regulation layer may not be completely captured, using the complemented “natural null mutant” *H. volcanii* model.

Our temporal analyses of *H. salinarum* dRNA-seq data revealed cases of differential processing during growth. Although the vast majority of TPS breakpoint signals follow the same pattern of the overall gene, i.e., increased signal when the overall gene expression signal increases and vice versa, a few cases display opposite patterns, i.e., decrease in transcript abundance at TPS breakpoint while the cognate genes increase expression and vice versa (Figure S5 and Figure 6). We highlighted *ftsZ2*, a gene involved in cell division, which increased its expression from exponential to stationary phases and at the same time decreased transcript cleavage at the conserved TPS breakpoint (Figure 5). The elongation factor *eEF1A*, on the other hand, showed a negligible decrease in expression levels as *H. salinarum* grew, but a marked breakpoint at the conserved TPS position, indicating an additional post-transcriptional effort to stop translation. Other non-conserved TPS exist, showing differential regulation (even within these highlighted examples) but we kept our focus on those shared between *H. salinarum* and *H. volcanii*. The source of this analysis is available, and growth-dependent post-transcriptional regulation candidate events can be explored in the interactive browser made available as supplemental data.

*H. salinarum* ribosome footprinting [35] allowed us to notice a trend between concentration of ribosome-bound RNA fragments and TPS breakpoints (Figure S6 and Figure 7). Often, we find colocalization between TPS and Ribo-seq local peaks (64% of conserved TPS). It is known from the Ribo-seq analysis field that the signal profile reflects the dynamics of an array of ribosomes translating an mRNA [59,60]. Internal signal peaks are often interpreted as ribosome pauses. We are not able to resolve whether the processing event pointed by the TPS is the following: (i) some sort of response to paused/stalled ribosomes, such as “No-Go” decay (NGD) [51,61]; or (ii) products of mRNA precursors interacting/interfering with ribosomes [62]. In both cases, local enrichment peaks are expected in Ribo-seq signals.

Finally, we kept advancing the understanding of ncRNAs associated with insertion sequences (IS). Our research group characterized a set of transcripts near the 3′ end of transposable elements belonging to the IS*1341* group [52]. These transcripts overlap the IS coding regions in the same orientation, harbor a strong predicted secondary structure signature and show expression patterns distinct from their cognate gene (often anti-correlated) in several experimental conditions. The IS*1341* group belongs to a very ancient archaeal transposable element family, IS*200/*IS*605*, and conservation of these discovered sense overlapping transcripts (sotRNAs) granted the establishment of two new RNA families in the Rfam database v14.4 [63], sot0042 and sot2652 (Rfam accessions: RF02656 and RF02657, respectively). The TPS mapping carried out here showed that transcription start sites (TSS) of sotRNAs are, in fact, processing sites (TPS) and, therefore, these ncRNAs are derived from larger precursors. Our observations are not limited to Haloarchaea but rather can also be detected in Thermococci and even in bacteria. Since the level of nucleotide sequence conservation among all these bacterial and archaeal organisms upstream or downstream of the TPS is very low, the interpretation of such positional coincidence with the transposase domain is still elusive to us. Taken together, these results allow us to recognize that sotRNAs, with sot0042 and sot2652 RNA families, being only particular cases, are processing products from harboring IS transcripts.

Future steps of this work include in silico and experimental efforts. It would be important to apply the straightforward dRNA-seq “inversion” to other organisms, including non-halophiles. Additional organisms are continuously being considered and results are made available at http://labpib.fmrp.usp.br/~rvencio/tpsdraft/ (accessed on 27 May 2021). Experimental follow-ups on post transcriptional regulation predictions, such as for the physiologically relevant gas vesicle system (Figure S2) or FtsZ2 cell division system (Figure 5), must be carried out at protein level to confirm decoupling between the RNA message and protein action in halophiles. High-throughput proteomics is the natural choice to search and validate post-transcriptional regulatory mechanisms pointed here. Structural (not sequence-based only)-motif finding guided by TPS (Figure 4) and RNase deletions may reveal post-transcriptional regulatory circuits.

## 5. Conclusions

Using an in silico comparative transcriptomics approach, we maximized the data value by reanalyzing published RNA-seq variants to gain understanding on transcript processing. We mapped transcript processing sites (TPS) in *H. salinarum* NRC-1 and *H. volcanii* DS2, and drew correlations with *H. salinarum* differential expression, RNase deletion and ribosome footprint data, especially for TPS conserved between both halophilic species. The interactive map provided as supplemental files can pave the way for experimental validation of some biological hypotheses raised in this work. Our intent is to provide a useful platform for the halophilic microorganism research community.

## Data Availability

Not applicable.

## Supplementary Materials

The following are available online at https://www.mdpi.com/article/10.3390/genes12071018/s1, Figure S1: Genes with highest TPS density in *H. salinarum* and *H. volcanii.* Figure S2: Experimental validation of predicted TPS. Figure S3: Example of internal TPS in salt regulation gene. Figure S4: Putative signature found in dysregulated genes after VNG2099C RNase deletion. Figure S5: Example of differential processing at TPS during *H. salinarum* growth. Figure S6: Example of translation affecting probably due to TPS during *H. salinarum* growth. Figure S7: Example of conserved TPS, Ribo-seq and rancRNA signal coincidence in *H. salinarum* and *H. volcanii*. Figure S8: Identification of processing site in sense overlapping transcripts VNG_sot0013 and VNG_sot2652. Figure S9: Identification of processing sites in sense overlapping transcripts in *T. kodakaraensis* IS*605* insertion sequence family. Figure S10: Identification of processing site in IS associated sense overlapping transcripts in Bacteria. Table S1: Transcript processing sites (TPS) identified in *Halobacterium salinarum* NRC-1. Table S2: Transcript processing sites (TPS) identified in *Haloferax volcanii* DS2. Table S3: Non-filtered TPS search in *H. salinarum* NRC-1. Table S4: Non-filtered TPS search in *H. volcanii* DS2. Table S5: Transcript processing sites (TPS) conserved between *H. salinarum* NRC-1 and *H. volcanii* DS2. Table S6: Gene Ontology functional enrichment analysis of genes with conserved TPS. Table S7: Internal TPS present in *H. salinarum* with no correspondent in *H. volcanii*. Table S8: Internal TPS present in *H. volcanii* with no correspondent in *H. salinarum*. Table S9: Antisense transcript processing sites (aTPS) identified in *H. salinarum.* Table S10: Antisense transcript processing sites (aTPS) identified in *H. volcanii*. Table S11: TPS and aTPS pairs in *H. salinarum*. Table S12: TPS and aTPS pairs in *H. volcanii*. Table S13: Conserved TPS co-localized with local Ribo-seq peaks in *H. salinarum*. Table S14: Putative conserved rancRNAs between *H. salinarum* and *H. volcanii*. File S1: Gaggle Genome Browser file with dRNA-seq data and TPS positions in *H. salinarum* NRC-1. File S2: Gaggle Genome Browser file with dRNA-seq data, rancRNA and TPS positions in *H. volcanii* DS2. File S3: GGB file with TEX− data at exponential and stationary phases in *H. salinarum*. File S4: GGB file with Ribo-seq data and conserved TPS in *H. salinarum*.
