# Supplementary material for: Halobacterium salinarum and Haloferax volcanii Comparative Transcriptomics Reveals Conserved Transcriptional Processing Sites
Source: Genes (Basel). 2021 Jun 30;12(7):1018. doi: 10.3390/genes12071018 (PMC8303175; doi:10.3390/genes12071018)
Supplement: Supplementary file 1 [file genes-12-01018-s001.zip › Amr_et_al_genes_2021_v39-zipfile/Figure_S4_v30.pdf]

Figure S4

(a)

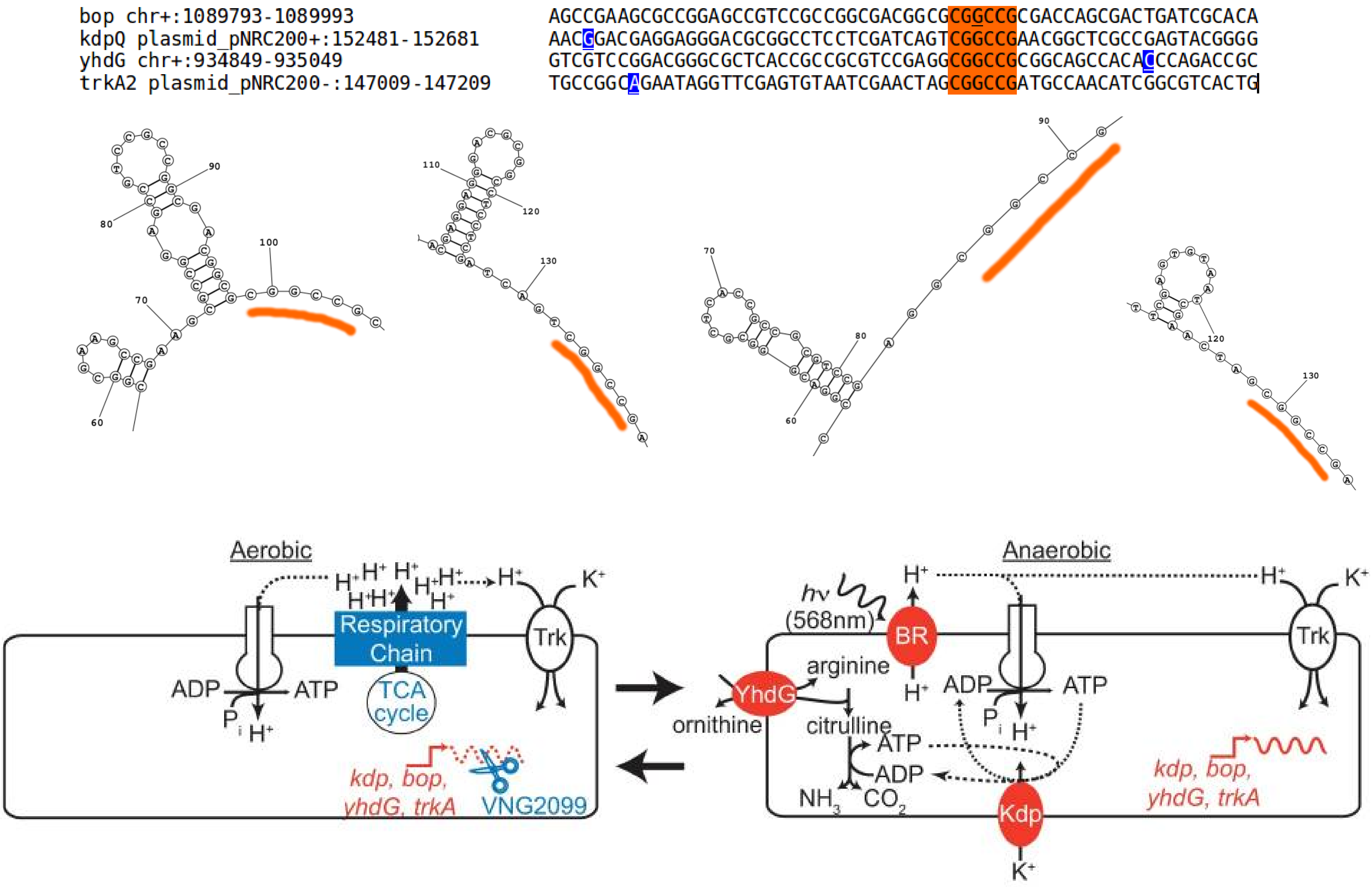

bop chr+:1089793-1089993 AGCCGAAGCGCCGGAGCCGTCCGCCGGCGACGGCGCGGCCGCGACCAGCGACTGATCGCACA
kdpQ plasmid_pNRC200+:152481-152681 AACGGACGAGGAGGGACGCGGCCTCCTCGATCAGTCGGCCGAACGGCTCGCCGAGTACGGGG
yhdG chr+:934849-935049 GTCGTCCGGACGGGCGCTCACCGCCGCGTCCGAGGCGGCCGCGGCAGCCACACCCAGACCGC
trkA2 plasmid_pNRC200-:147009-147209 TGCCGGCAGAATAGGTTCGAGTGTAATCGAACTAGCGGCCGATGCCAACATCGGCGTCACTG
Aerobic
Respiratory Chain
TCA cycle
ADP
ATP
Trk
kdp, bop, yhdG, trkA
VNG2099
Anaerobic
hv (568nm)
BR
YhdG
arginine
ornithine
citrulline
ATP
ADP
NH3
CO2
Kdp
Trk
kdp, bop, yhdG, trkA

(b)

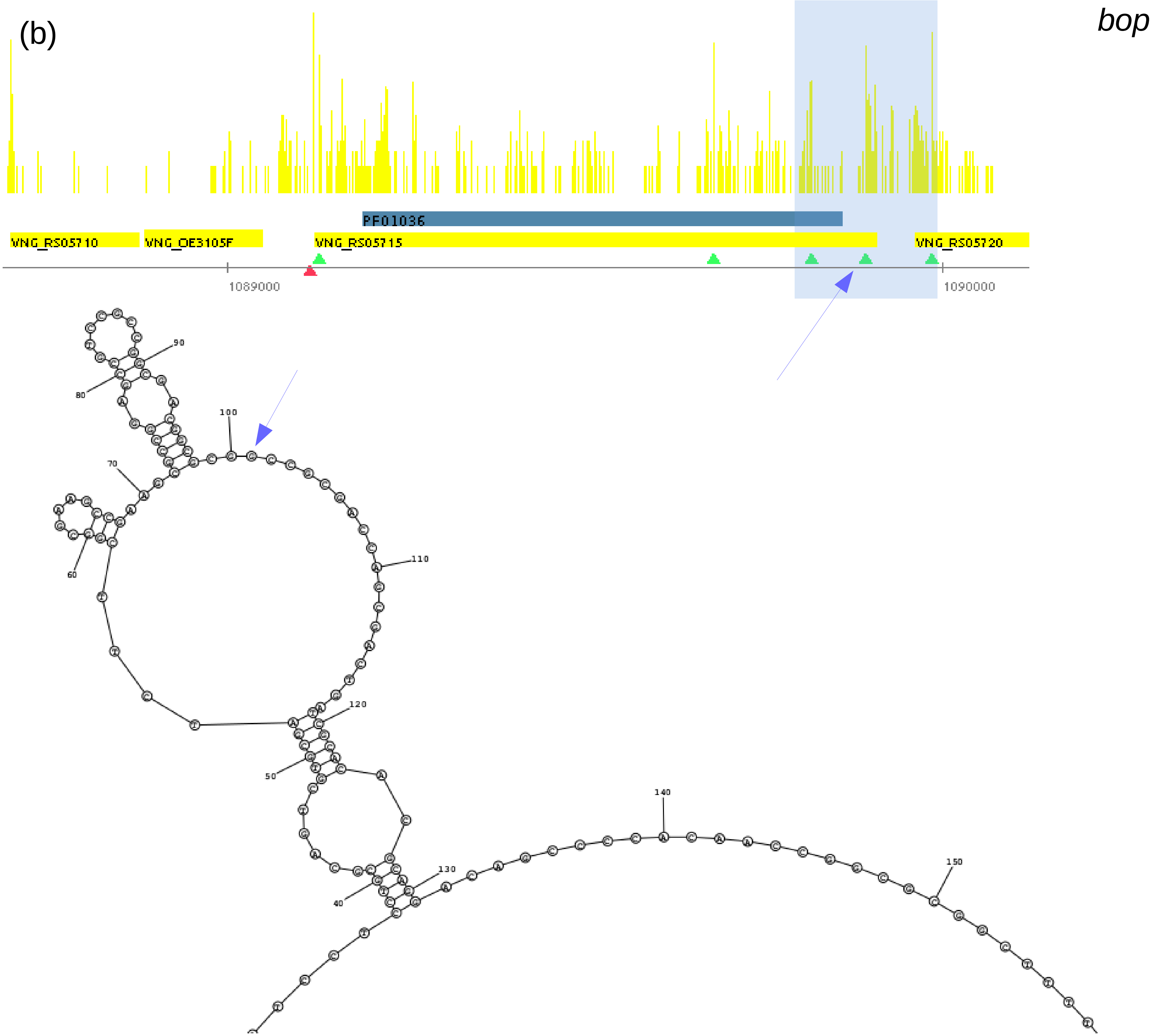
bop
PF01036
VNG_RS05710
VNG_OE3105F
VNG_RS05715
VNG_RS05720
1089000
1090000
40
50
60
70
80
90
100
110
120
130
140
150

(c)

kdpQ

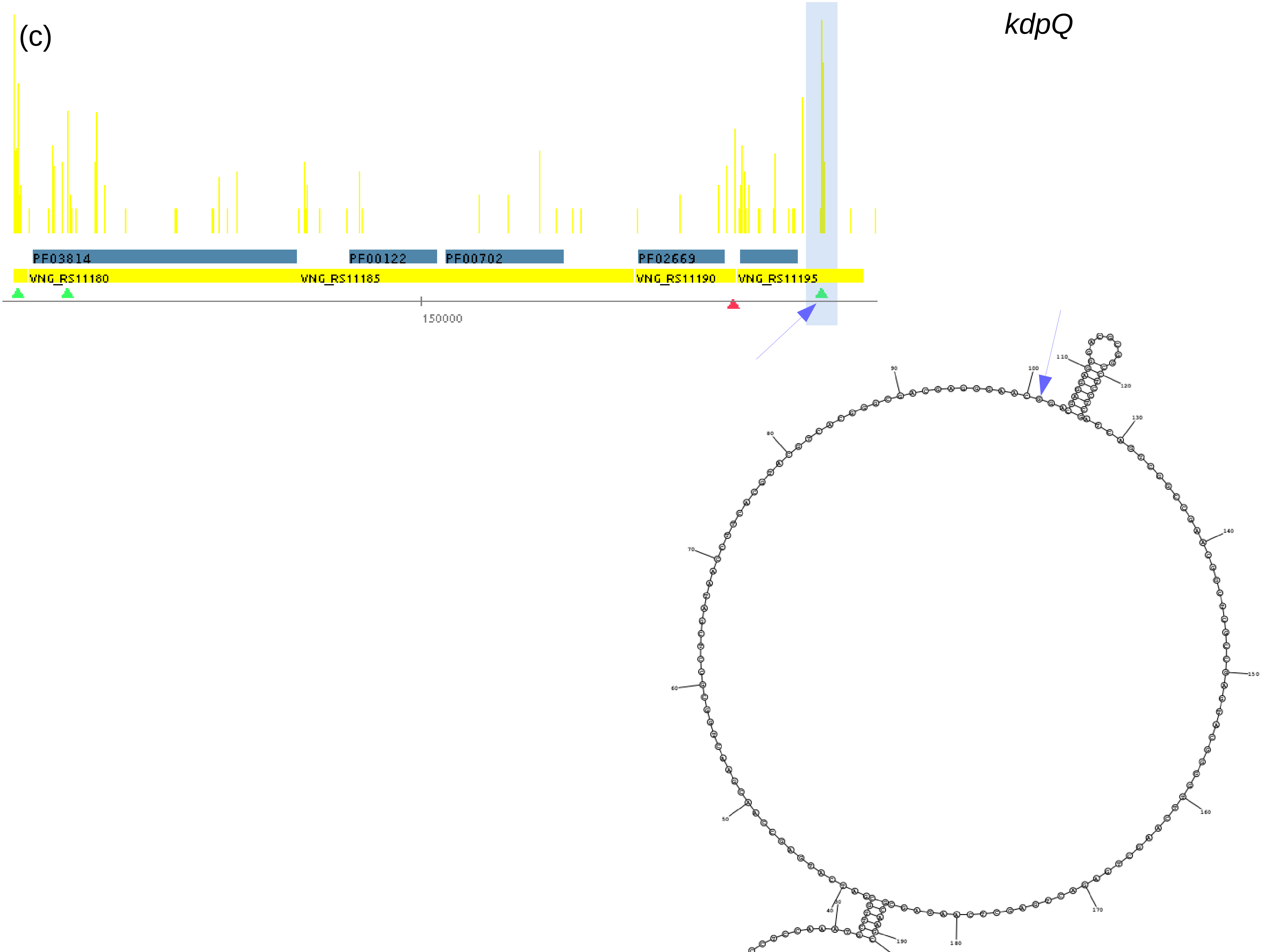
PF03814
PF00122
PF00702
PF02669
VNG_RS11180
VNG_RS11185
VNG_RS11190
VNG_RS11195
150000

(d)

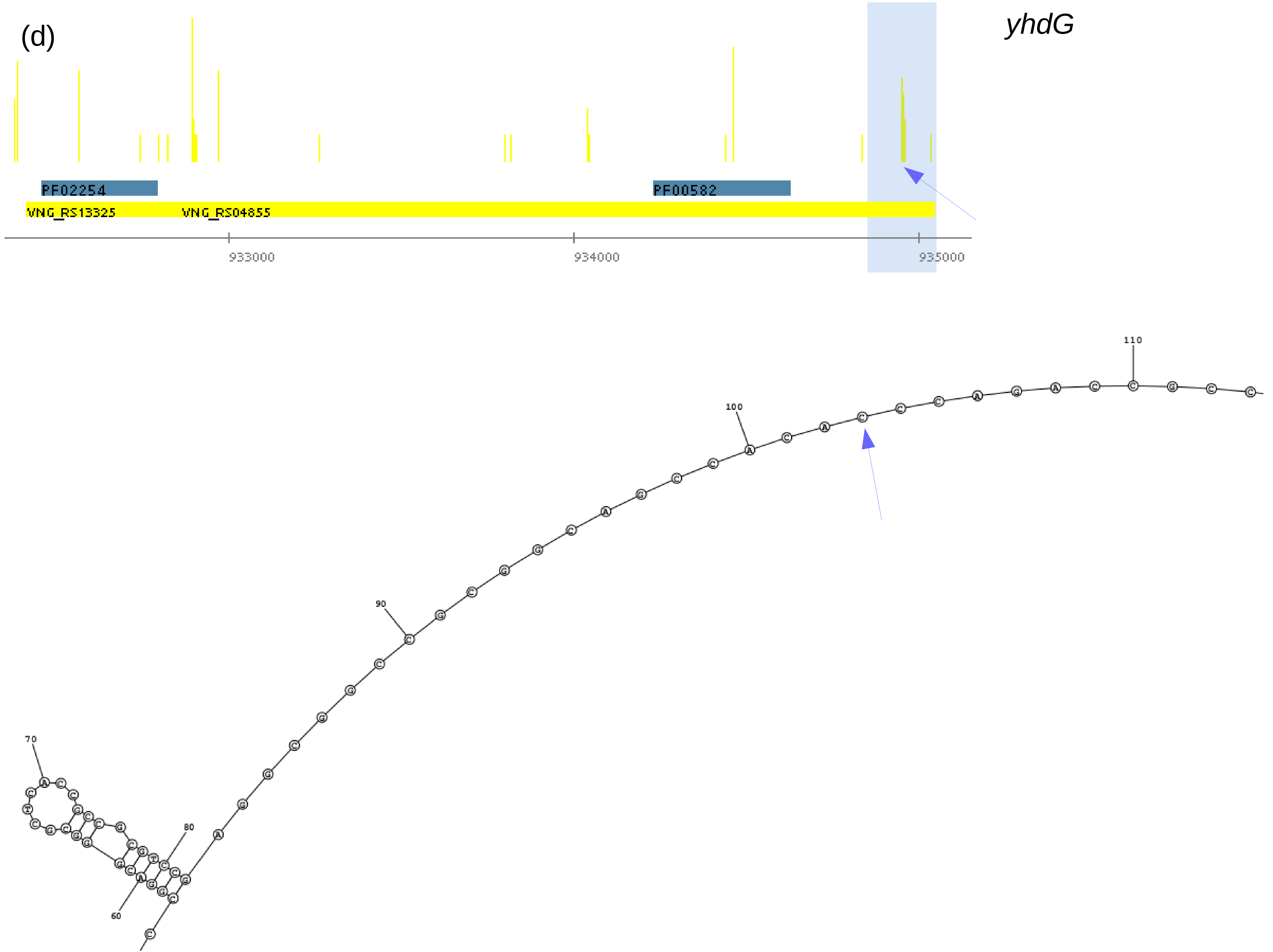
yhdG
PF02254
PF00582
VNG_RS13325
VNG_RS04855
933000
934000
935000
60
70
80
90
100
110

(e)

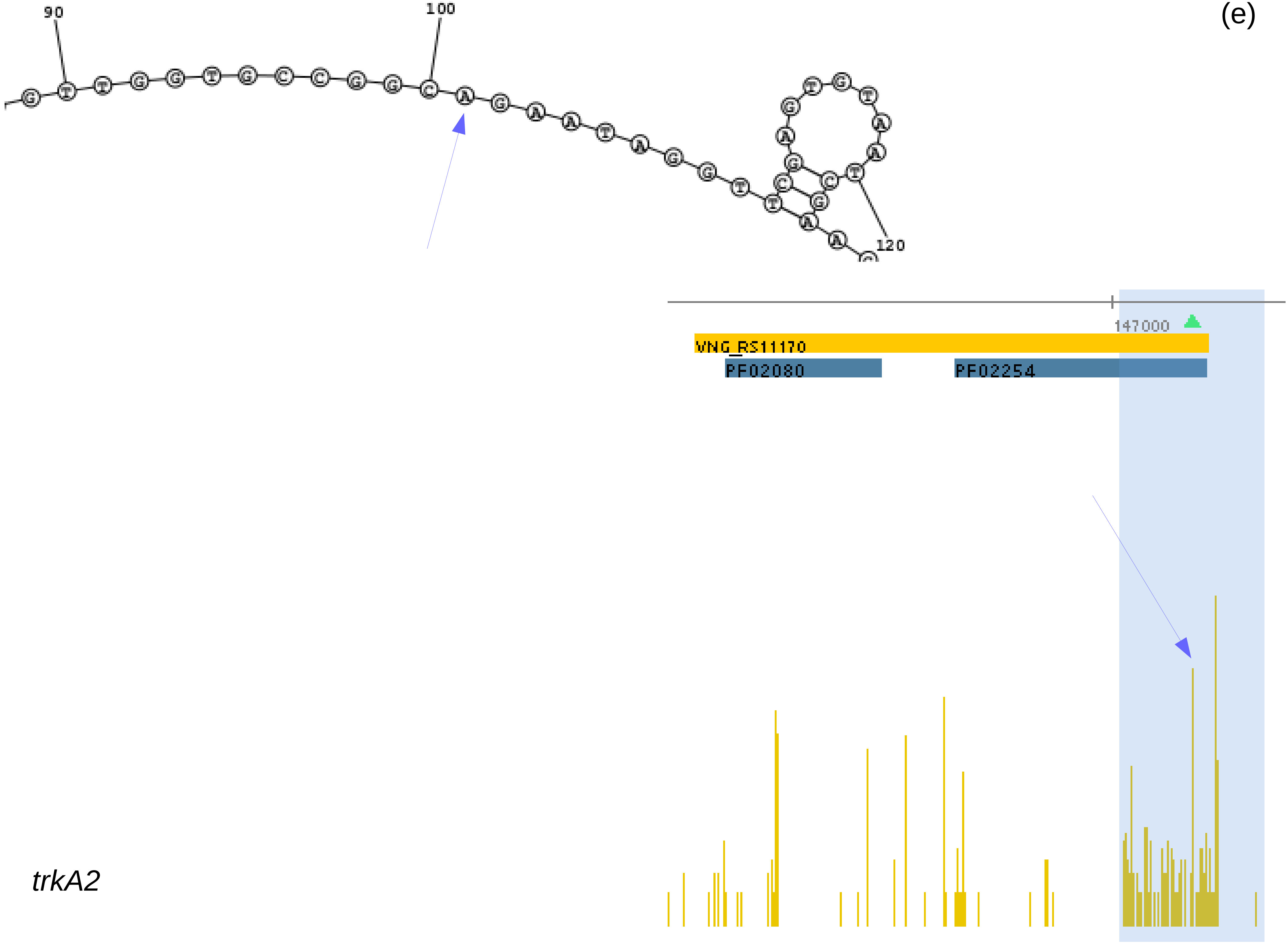

trkA2
