# Supplementary material for: Halobacterium salinarum and Haloferax volcanii Comparative Transcriptomics Reveals Conserved Transcriptional Processing Sites
Source: Genes (Basel). 2021 Jun 30;12(7):1018. doi: 10.3390/genes12071018 (PMC8303175; doi:10.3390/genes12071018)
Supplement: Supplementary file 1 [file genes-12-01018-s001.zip › Amr_et_al_genes_2021_v39-zipfile/amr_TPS_genes_LEGEND_suppl_figures_v02.pdf]

Article

Halobacterium salinarum and *Haloferax volcanii* comparative transcriptomics reveals conserved transcriptional processing sites

Supplemental Figures Legends

Figure S1 - Genes with highest TPS density in *H. salinarum* and *H. volcanii*.
(a) RNase H domain containing exoribonuclease (VNG_RS04745 *locus*) presents the highest TPS density in *H. salinarum*, 8 TPS (green triangles) in a 200 nt window (light blue highlight). Distribution of relative numbers of aligned reads starting at a given genomic coordinate in TEX- libraries is shown in yellow (arbitrarily log-scaled normalized counts). Pfam domain annotation (blue rectangle) and coding sequences are in forward strand (yellow rectangle) thus 5'→3' direction is left to right. Red triangle marks ChIP-seq based binding site of TfbD transcription factor co-localized with genes' TSS (from Wilbanks *et al.*, 2012). 5' UTR is highly processed.
(b) "cold-shock" *cspA4* gene (HVO_RS14265 *locus*) present the highest TPS density in *H. volcanii*, 11 TPS (green triangles) in a 200 nt window (light blue highlight). Coding sequence is in reverse strand (orange rectangle) thus 5'→3' direction is right to left.

Figure S2 - Experimental validation of predicted TPS.
(a) Experimental validation of predicted TPS using gene *cspA1* (VNG_RS00395 *locus*). Consecutive TPS (green triangles) predict mRNAs of different sizes (arrows) from TSS to TPS_8361_1 (#4), TPS_8363_1 (#3), TPS_8365_1 (#2) and TPS_8366_1 (#1). Northern blot probe location is marked by the black rectangle. Right panel shows a northern blot published by our group (Zaramela *et al.*, 2014) with bands consistent with transcripts cleaved at TPS sites. Lower panel shows secondary structure prediction for the larger transcript and each processing site location (numbered arrows).
(b) Upper panel adapted from Figure 4C in Ruepp & Soppa (1996) where two-headed arrows mean inexact transcript boudaries in *H. mediterranei*. Lower panel shows an histogram using *H. salinarum* data. Distribution of relative numbers of aligned reads starting at a given genomic coordinate in TEX- and TEX+ libraries, shown in yellow and blue, respectively (arbitrarily log-scaled normalized counts). Pfam domain annotation (blue rectangle) and coding sequences are in reverse strand (orange rectangle) thus 5'→3' direction is right to left. Green arrows point to the TPS-transcript correspondence: TPS_20943_1 and TPS_19346_1. Green triangles are TPS defined in the present work (see Supplemental File 1 to navigate them). Red triangle marks ChIP-seq based binding site of TfbD transcription factor co-localized with genes' TSS (from Wilbanks *et al.*, 2012).
(c) Conservation of TPS between *H. salinarum* and *H. mediterranei* explains the operon fragment observed in *H. mediterranei*. Upper panel taken directly from Jäger *et al*. (2002) with arrows representing observed transcripts in *H. mediterranei*. Lower panel shows *H. salinarum* dRNA-seq data. Operon representations are shown schematically approximately at scale aligned by CDS (rectangles) and TSS (blue arrow). Distribution of relative numbers of aligned reads starting at a given genomic coordinate in TEX- libraries is shown in yellow (arbitrarily log-scaled normalized counts). The *gvpDEFGHIJKLM* operon is in reverse strand in both organisms thus 5'→3' direction is right to left. The TPS which would create an equivalent of *H. mediterranei*'s *gvpDE'* (2.0 kb) in *H. salinarum* is TPS_19964_1 (green arrow).
(d) TPS in *gvpA1*, encoding the most important structural protein in the gas vesicle system (VNG_RS10625). TPS (TPS_18335_1, green arrow) is located at the basis of a strong stem-loop structure involved in transcript stability. CDS are represented by yellow rectangles in forward strand (5'→3' is left to right), Pfam domain annotations are denoted by blue rectangles. Distribution of relative numbers of aligned reads starting at a given genomic coordinate in TEX- and TEX+ libraries are shown as yellow and blue vertical lines, respectively (arbitrarily log-scaled normalized counts). Light blue highlights along genome coordinates denote the actual sub-sequence selected for detailed secondary structure prediction.
Predicted structures are colored according to base pair probabilities depicted in nearby graphical scales. All structures were predicted using RNAfold web server (Gruber et al., 2008) using default parameters except energy parameters which were set to "Turner model, 1999".

Figure S3 – Example of internal TPS in salt regulation gene.
(a) Example of transcript processing site (TPS) signal in *H. salinarum* dRNA-seq data. This is the sodium transporter *kef1* gene (VNG_RS07995 *locus*). Pfam domain annotation (blue rectangle) and coding sequences are in reverse strand (orange rectangle), thus 5’→3’ direction is right to left. Distribution of relative numbers of aligned reads starting at a given genomic coordinate in TEX- libraries is shown in orange (arbitrarily log-scaled normalized counts). Aligned reads coverage along genomic coordinates for TEX+ and TEX- are shown in dark green and light green, respectively (absolute counts normalized and arbitrarily scaled). Magenta arrow points to the identified site (TPS_13995_1) along with typical TEX+ depletion signature. All possible archaeal start/stop codons in frame with *kef1* are shown in the bottom as vertical tick marks (ATG highlighted and stop codons in red).
(b) Regular RNA-seq coverage profile of kef1 gene in *H. walsbyi*. Coverage profile of RNA-seq alignments (orange) are shown using length-normalized coordinates (D = 0 at start codon, D = 100 at stop codon inside HQ_RS10745 *locus*). Transcript abundance peak highlighted at D = 71 is positionally equivalent to TPS found in *H. salinarum*. Light and dark blue marks delimit the same Pfam domains as in (a).
(c) Putative *Natrinema* sp. J7-2 TPS inside the sodium transporter *kef1* CDS. (NJ7G_RS00730 *locus*, reverse strand, 5’→3’ is right to left). Normalized read alignment coverage is shown for low (15% NaCl, light blue), optimal (25% NaCl, blue) and high (30% NaCl, dark blue) salt concentrations along genomic coordinates only within CDS boundaries. RNA-seq data indicates that *kef1* is differentially processed at this site depending on salt concentration. The putative processing site is marked by a vertical dotted line at relative position D = 74. The expression level difference between the TPS-associated plateau and the overall gene is greater for low salt concentration (light blue vertical rectangle) and smaller for high salt concentration (dark blue vertical rectangle) although expression levels are higher in the optimal concentration (blue solid line profile).

Figure S4 – Putative signature found in dis-regulated genes after VNG2099C RNase deletion.

(a) Sequences from -100 to +100 around TPS (underlined bases) inside genes *bop*, *kdpQ*, *yhdG* and *trkA2* were used as alignment input. All genes presented a CGGCCG sequence (orange highlights) downstream of a strong stem-loop. The secondary structure predictions were filtered to report only base pairings with >0.99 probability. RNase-mediated phenotypic switching scheme was adapted directly from Wurtmann et al. (2014). Zoom out of all four predicted structures are shown in (b) to (e).

(b) Overview of *bop* transcript secondary structure prediction result. Sequences that do not base pair with anything were cropped for clarity. Arrows point to TPS position. Light blue highlights the actual 100+1+100 nt sequence used for structure prediction. Pfam domain annotation (blue rectangle) and coding sequences (yellow rectangle) are in forward strand, thus 5'→3' direction is left to right. Distribution of relative numbers of aligned reads starting at a given genomic coordinate in TEX- libraries is shown in yellow (arbitrarily log-scaled normalized counts). Green triangles are TPS defined in the present work (see Supplemental File 1 to navigate them). Red triangle marks ChIP-seq based binding site of TfbD transcription factor co-localized with genes' TSS (from Wilbanks *et al.*, 2012).

(c) Overview of *kdpQ* gene, same data description as in (b).

(d) Overview of *yhdG*, same data description as in (b).

(e) Overview of *trkA2*, same data description as in (b), except that 5'→3' direction is right to left.

Figure S5 – Example of differential processing at TPS during *H. salinarum* growth.
(a) Growth curves from which original datasets were sampled. Left panel was addapted from Caten & Vêncio *et al*. (2018) Figure S1; dots show dRNA-seq data duplicate samples. Right panel was adapted from Lomana *et al*. (2020) Figure 7; lines show
(b) *eEF1A*, encoding an elongation factor (TPS_16108_1, VNG_RS10385). Pfam domain annotation (blue rectangle) and coding sequences are in reverse strand (orange rectangle) thus 5'→3' direction is right to left. Aligned reads coverage along genomic coordinates for TEX+ libraries at exponential and stationary phases are shown in light red (solid) and red (dots), respectively (log_2 counts normalized and arbitrarily jointly scaled). Blue arrows point to conserved TPS.
(c) a putative arsenic resistance operon repressor encoded at the VNG_RS03675 *locus* (TPS_2832_1). Coding sequence in forward strand (yellow rectangle) thus 5'→3' is left to right. Transcriptome signal same as (b).
(d) *pcn*, encoding a DNA polymerase III subunit (TPS_14733_1, VNG_RS08800). Coding sequence is in reverse strand (orange rectangle) thus 5'→3' direction is right to left. Light blue highlight delimits the sub-sequence used for secondary structure prediction. Transcriptome signal same as (b).

Figure S6 – Example of translation affecting probably due to TPS during *H. salinarum* growth.
(a) The TPS (TPS_20943_1, vertical dashed line) is near the start codon inside *arcA* gene (zoomed in VNG_RS11635 *locus*) which encodes an arginine deiminase pathway gene. Coding sequence is in reverse strand (orange rectangle) thus 5'→3' direction is right to left and only first 600 bp are zoomed in out of ~1.5 kbp gene. Aligned reads coverage along genomic coordinates for TEX- libraries at exponential and stationary phases are shown in light red (solid) and red (dots), respectively (log_2 counts normalized and arbitrarily jointly scaled).
(b) *arcA* gene log_2 fold-change (M) between published multi-modality measurements in different time-points relative to the early exponential phase from Lomana *et al*. (2020) and Lorenzetti *et al*. (in prep) (Figure S5a, time-point 1: early exponential, 2: mid-exponential, 3: late exponential, 4: stationary, squares: RNA-seq data, triangles: Ribo-seq data).
(c) Secondary structure prediction, color coded by pairing probabilities, using 100 bp around TPS (green arrow) as input.

Figure S7 – Example of conserved TPS, Ribo-seq and rancRNA signal coincidence in *H. salinarum* and *H. volcanii*. Aligned reads coverage along genomic coordinates for ribosome footprint libraries (SRP119792, Lomana *et al*., 2020) are shown in gray (normalized counts arbitrarily scaled). Coding sequences are in reverse strand (5'→3' direction is right to left) or forward strand (5'→3' direction is left to right) if gene rectangles are orange or yellow, respectively. Pfam domain annotations are shown as blue rectangles with ID inside. Dark blue triangles point to conserved TPS. Magenta rectangles delimit published putative rancRNA *loci* in *H. volcanii* (Wyss *et al*., 2018).
(a) *pan1* gene (VNG_RS01995 and HVO_RS08770 *loci*) which encodes PAN-A proteasome-activating nucleotidase. Vertical light blue highlight shows the difference between *pan1* known alternative transcripts (Chamieh *et al*., 2008).
(b) VNG_RS04015 and HVO_RS20130 which encodes the putative archaeal translation factor aMBF1.
(c) VNG_RS04165 and HVO_RS12050, which encodes an archaeosortase, a system-associated glycotransferase.
(d) VNG_RS08100 and HVO_RS05870, which encodes a glutamine synthetase.
(e) VNG_RS09610 and HVO_RS05290, which encodes the 54 kDa protein of the signal recognition particle ribonucleoprotein complex.
(f) VNG_RS00020.
(g) VNG_RS04995. Gene *sdo1*, which encodes for a ribosome maturation protein.

Figure S8 – Identification of processing site in sense overlapping transcripts VNG_sot0013 and VNG_sot2652. Distribution of aligned reads starting at a given genomic coordinate (horizontal ruler) for TEX+ (blue) and TEX- (yellow) datasets. Vertical bars in blue and yellow are superimposed signals (arbitrarily scaled $\log_2$ of normalized counts) at the same position. Zoom in of coding sequences are in reverse strand (orange rectangle, *locus* ID inside, 5'→3' direction is right to left). Domain annotation (blue rectangle, PFAM ID inside). Green markers point to the TPS signatures which were not detected by the statistical significance finding methodology. **(a)** VNG_sot0013 and **(b)** VNG_sot2652 sotRNAs.

Figure S9 – Identification of processing site in sense overlapping transcripts in *T. kodakaraensis* IS605 insertion sequence family. Distribution of aligned reads starting at a given genomic coordinate (horizontal ruler) for TEX+ (blue) and TEX- (yellow) datasets. Vertical bars in blue and yellow are superimposed signals (arbitrarily scaled $\log_2$ of normalized counts) at the same position. Coding sequence is in forward strand (yellow rectangle, locus ID inside, 5'→3' direction is left to right). Green markers point to putative TPS inside *loci* **(a)** TK0298 and **(b)** TK1842.

Figure S10 – Identification of processing site in IS associated sense overlapping transcripts in Bacteria. Aligned reads coverage signal along *tnpB* gene coordinates for TEX+ (blue) and TEX- (yellow) datasets in:
(a) *Escherichia coli* K-12 (*locus* b1432),
(b) *Helicobacter pylori* 26695 (*locus* HP0989),
(c) *Streptomyces coelicolor* M145 (*locus* SCO3714),
(d) *Synechocystis* sp. PCC 6803 substr. GT-I (*locus* slr2062) and
(e) *Mycobacterium tuberculosis* H37Rv (*locus* Rv2978c).
Vertical dotted lines delimit the characteristic transposase DNA-binding protein domain OrfB_Zn_ribbon (PFAM database accession: PF07282).

© 2021 by the authors. Submitted for possible open access publication under the terms and conditions of the Creative Commons Attribution (CC BY) license (http://creativecommons.org/licenses/by/4.0/).
