# Supplementary figures and images for: Halobacterium salinarum and Haloferax volcanii Comparative Transcriptomics Reveals Conserved Transcriptional Processing Sites
Source: Genes (Basel). 2021 Jun 30;12(7):1018. doi: 10.3390/genes12071018 (PMC8303175; doi:10.3390/genes12071018)

# **Figure S10**

(a)

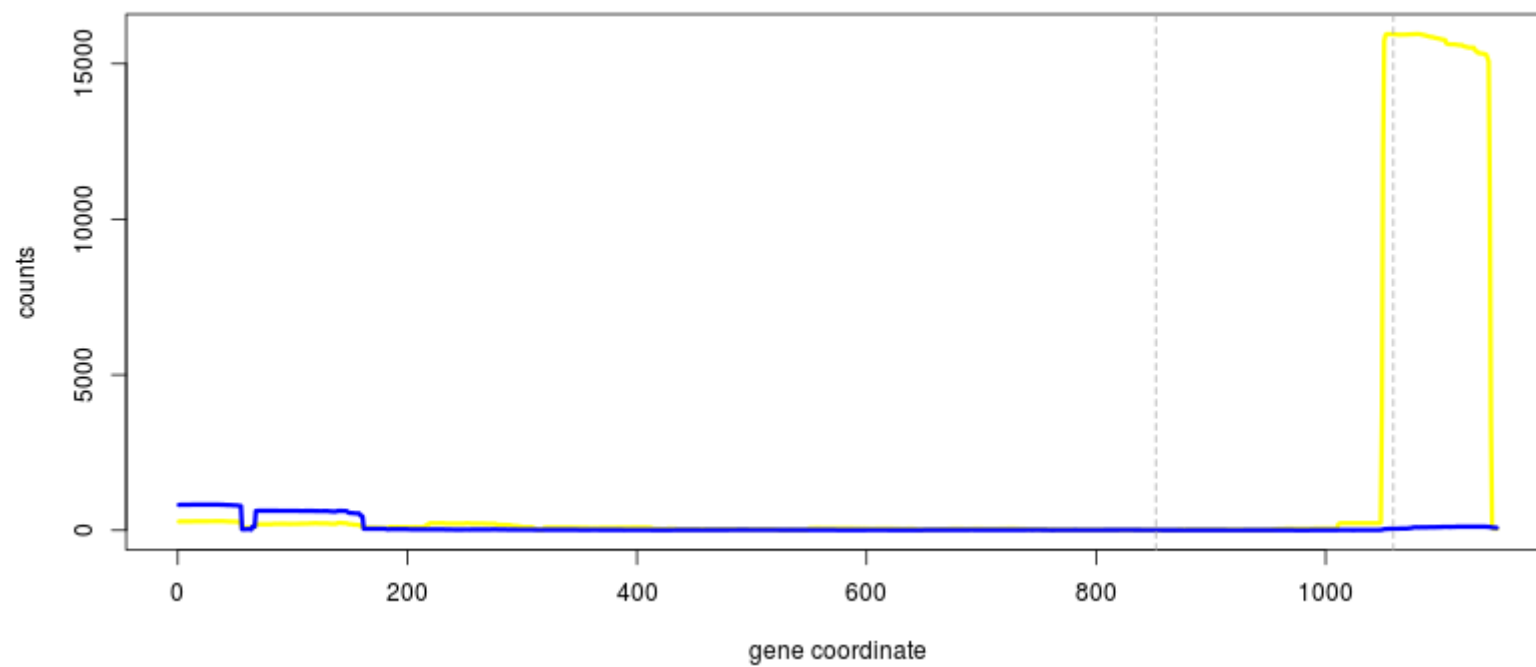

(b)

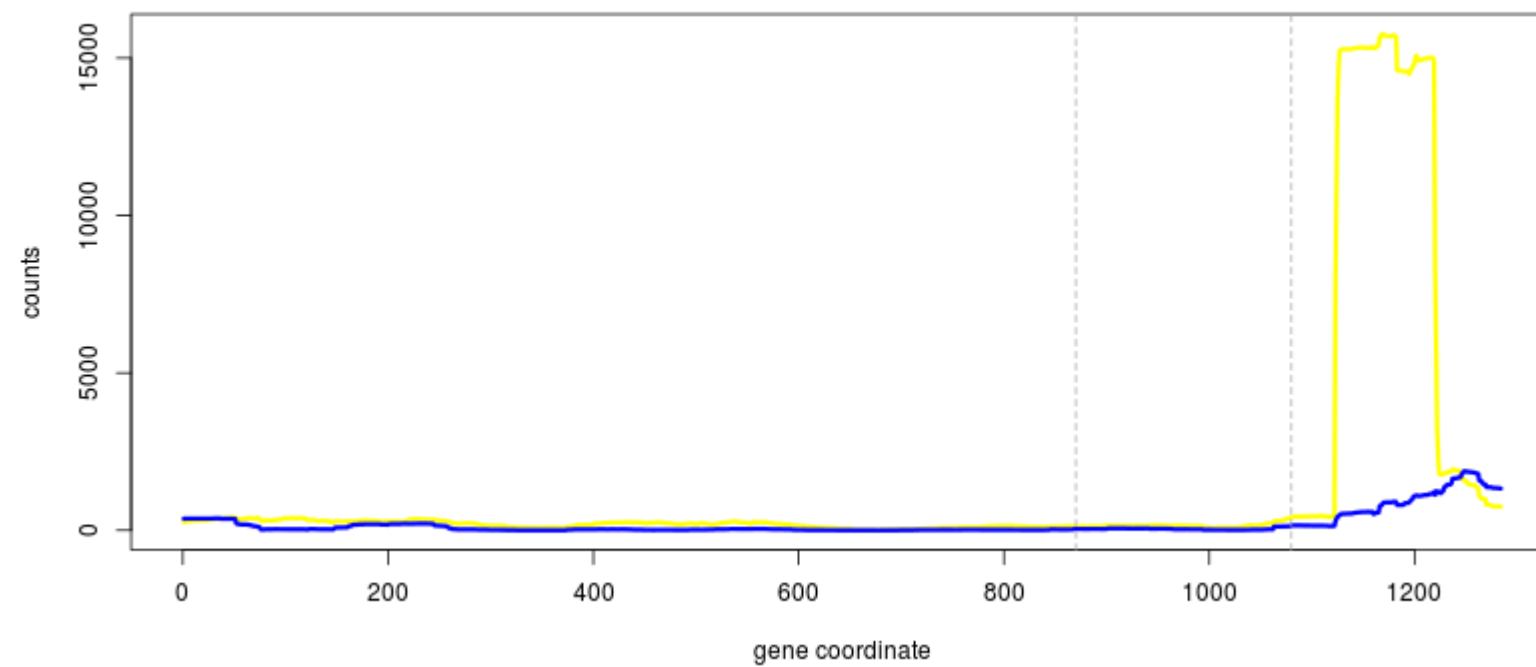

(c)

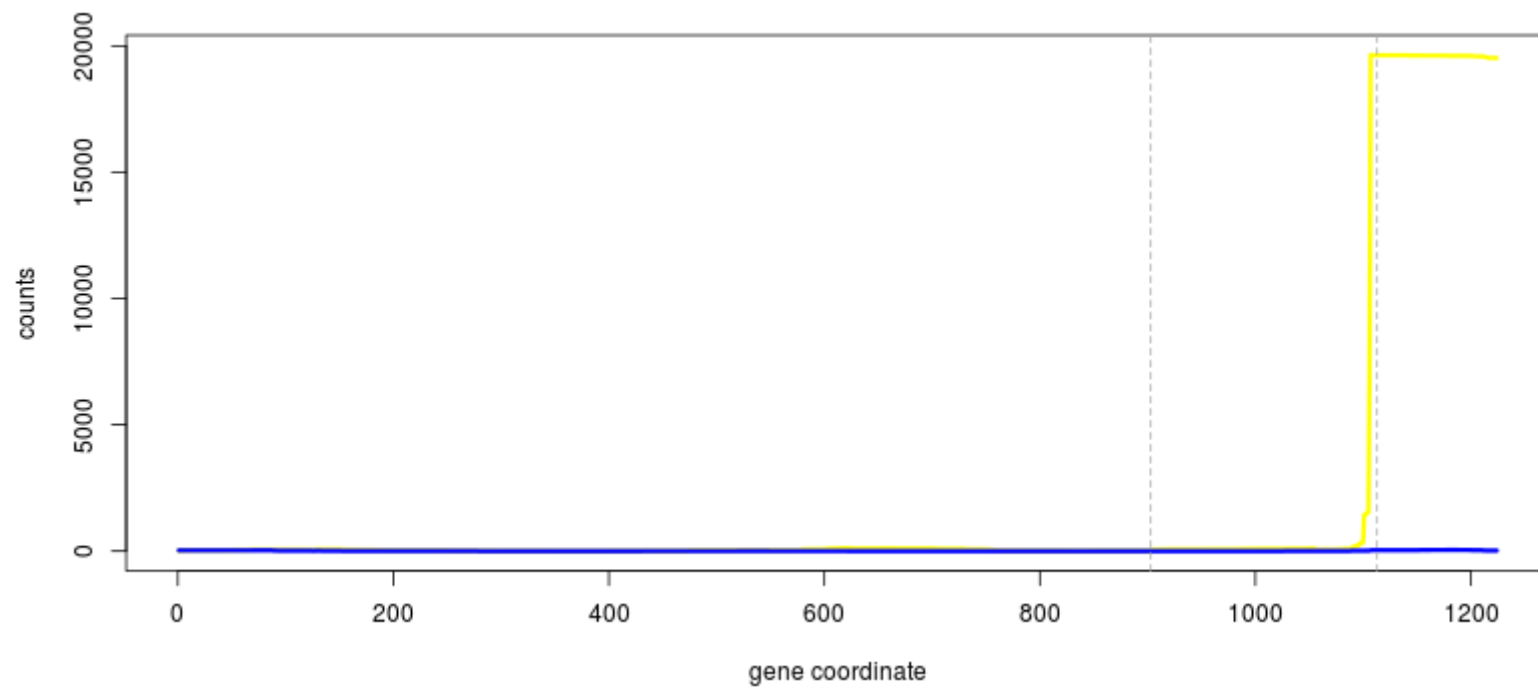

(d)

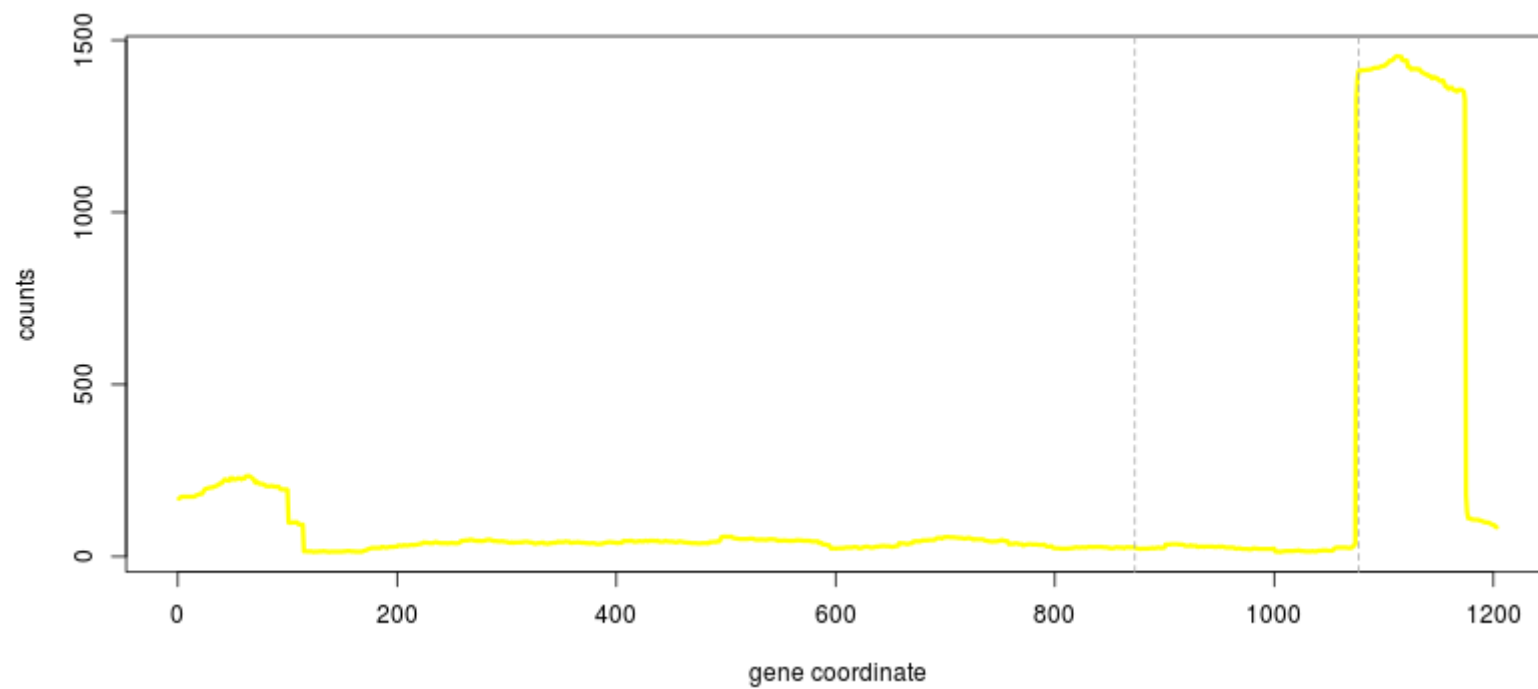

(e)

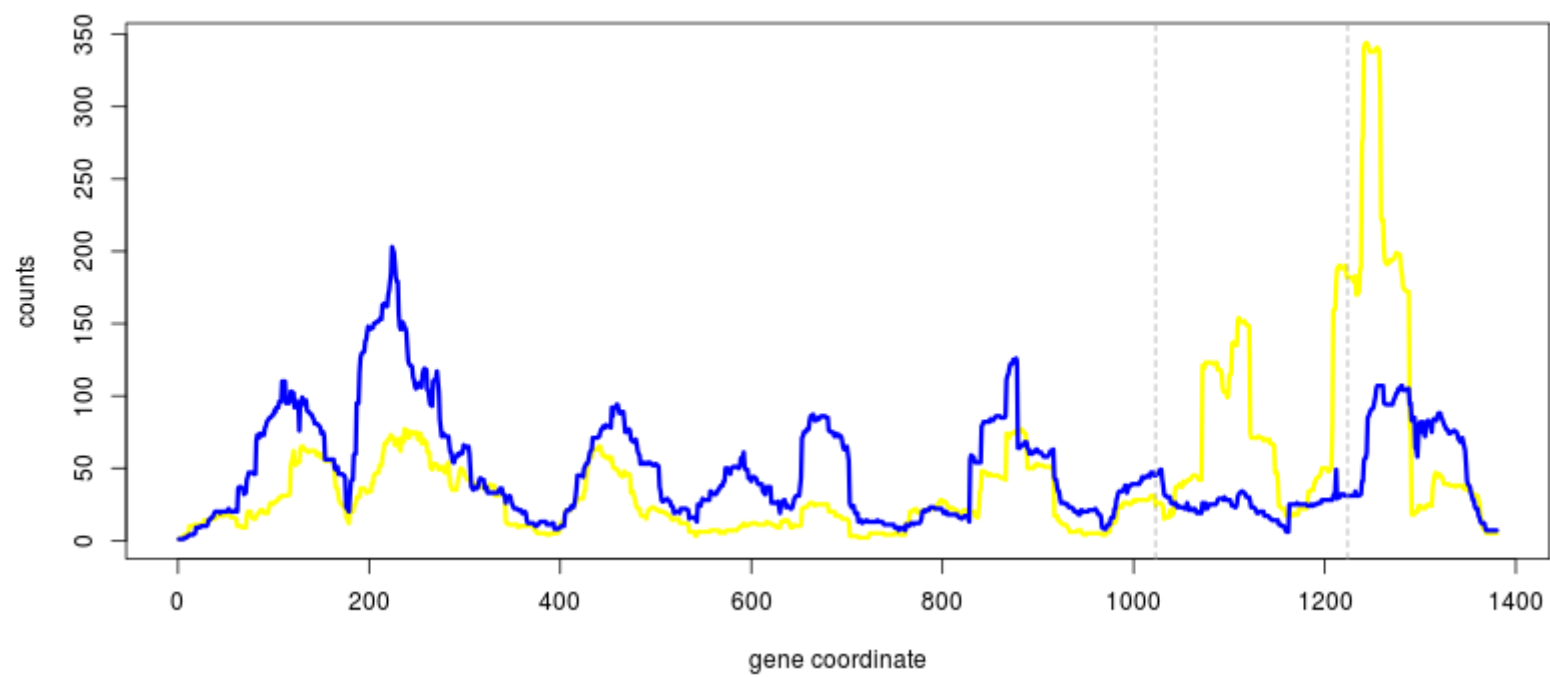

Supplement: Supplementary file 1 [file genes-12-01018-s001.zip › Amr_et_al_genes_2021_v39-zipfile/Figure_S10_v30.pdf]

# Figure S9

(a)

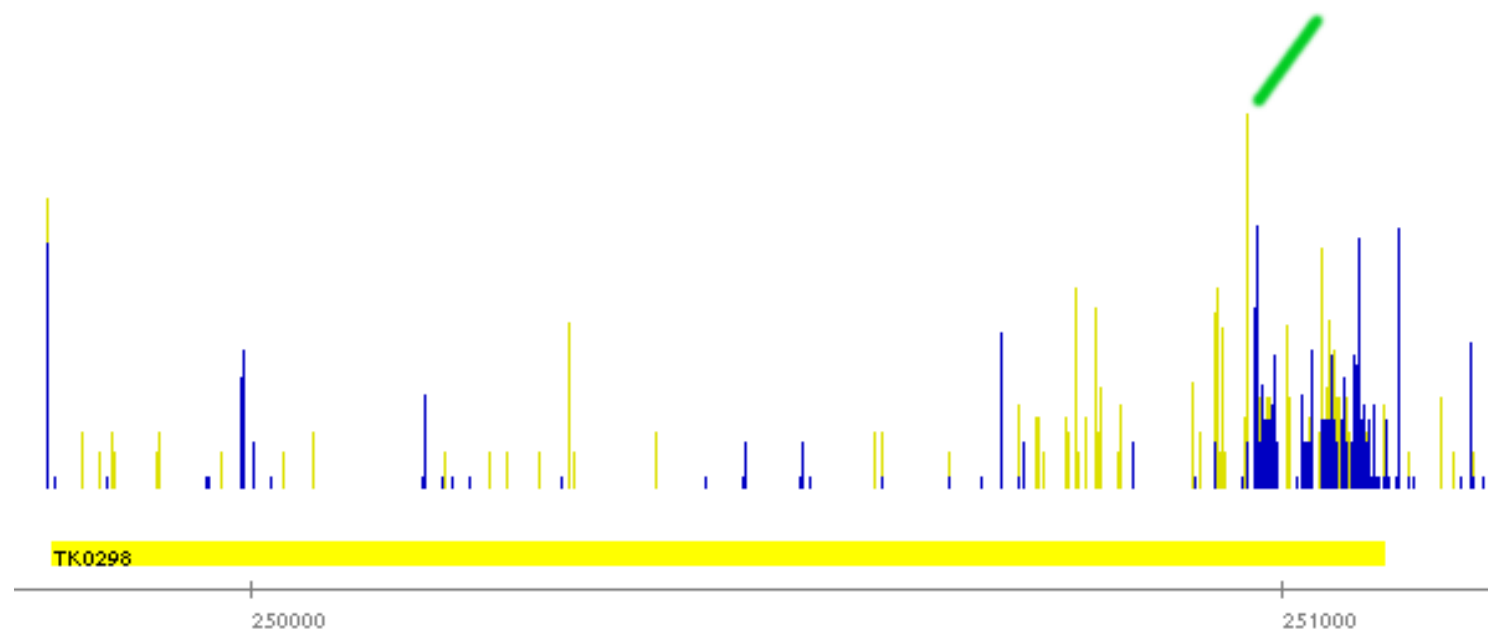

(b)

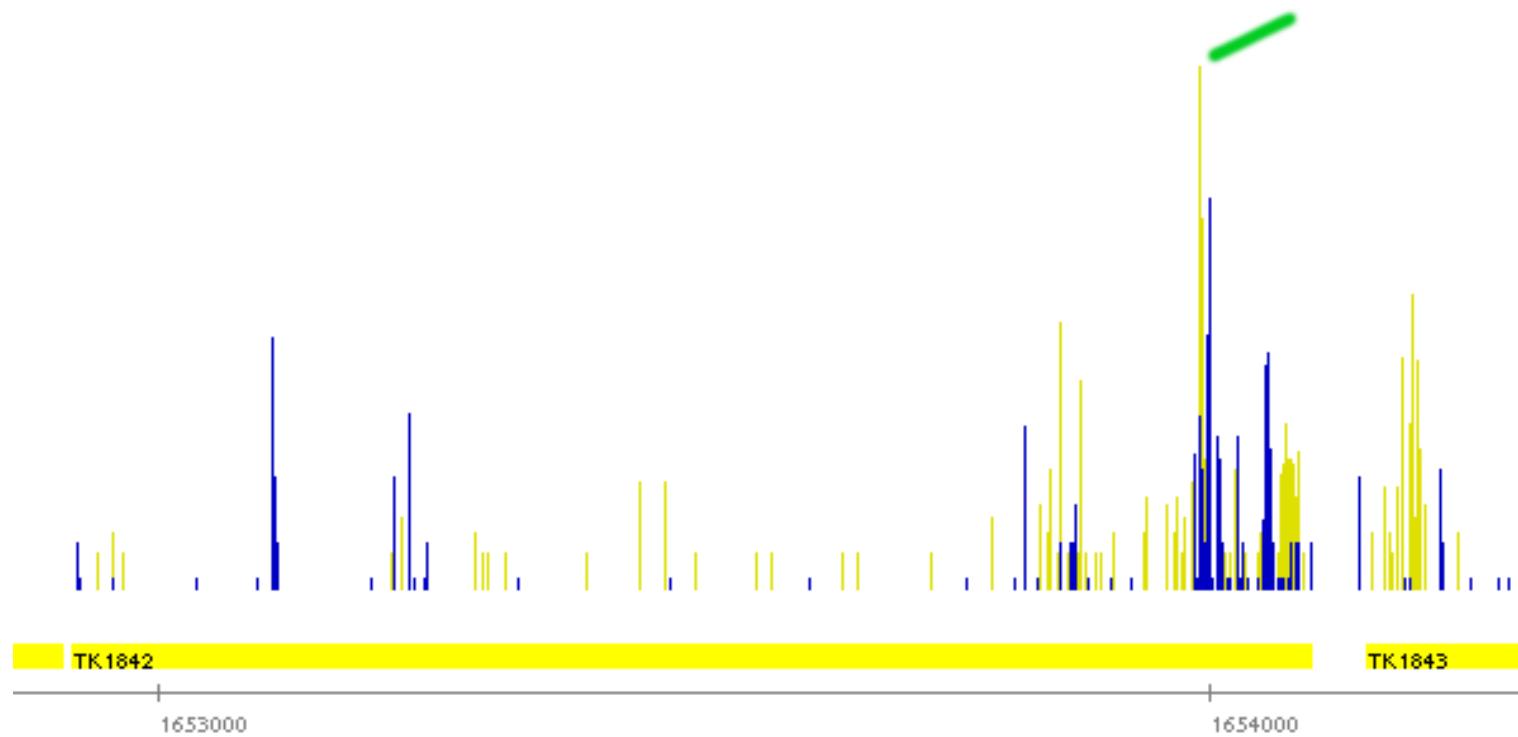

TK0298 and TK1842

Supplement: Supplementary file 1 [file genes-12-01018-s001.zip › Amr_et_al_genes_2021_v39-zipfile/Figure_S9_v30.pdf]

# Figure S8

(a)

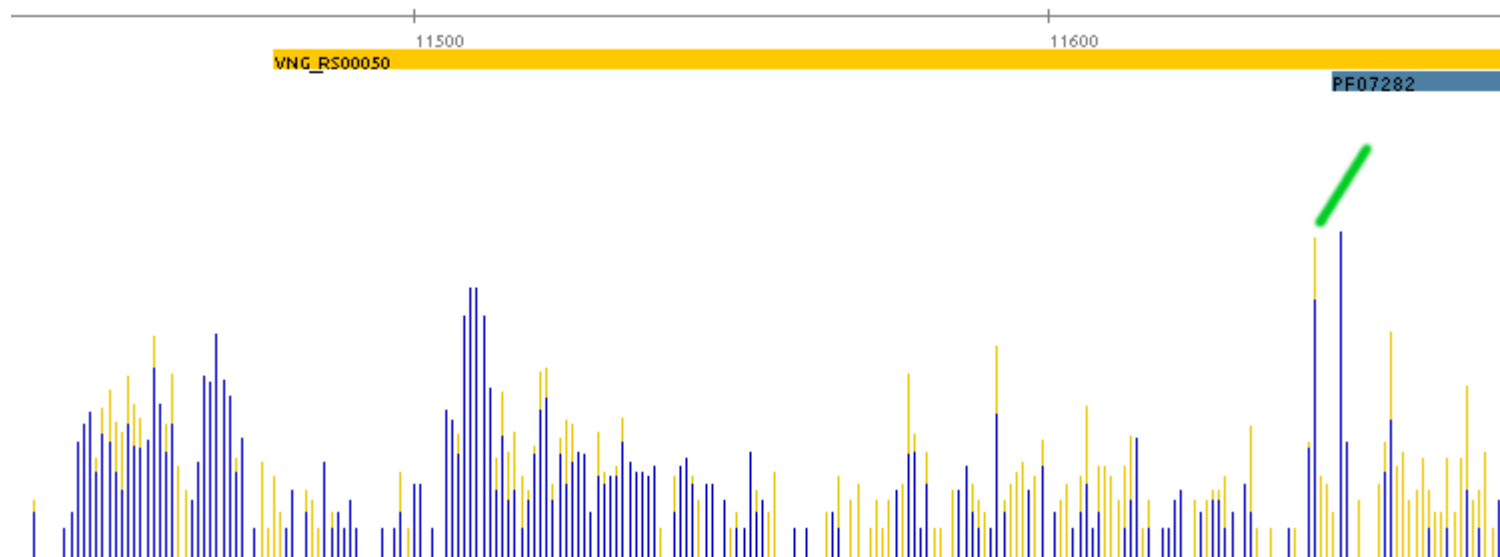

(b)

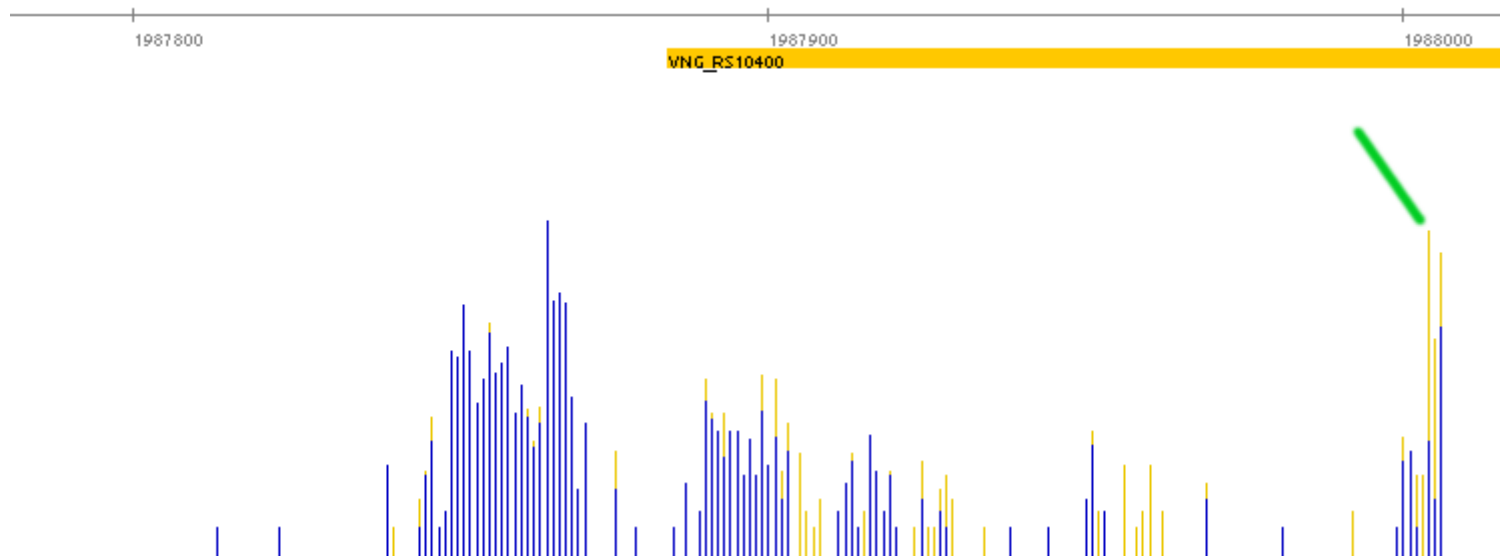

VNG\_sot0013 and VNG\_sot2652

Supplement: Supplementary file 1 [file genes-12-01018-s001.zip › Amr_et_al_genes_2021_v39-zipfile/Figure_S8_v30.pdf]

# Figure S7

(a)

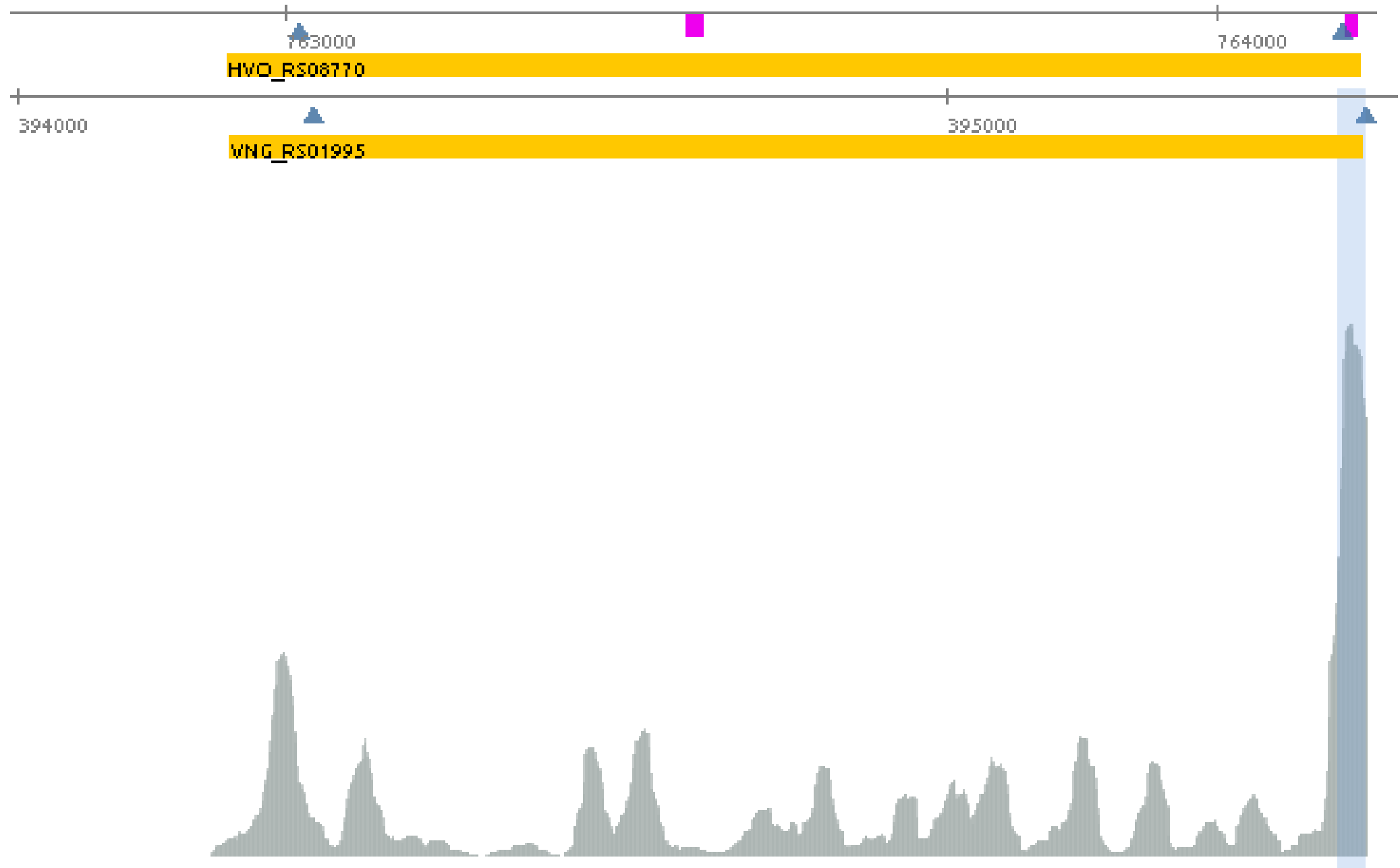

VNG\_RS01995

(b)

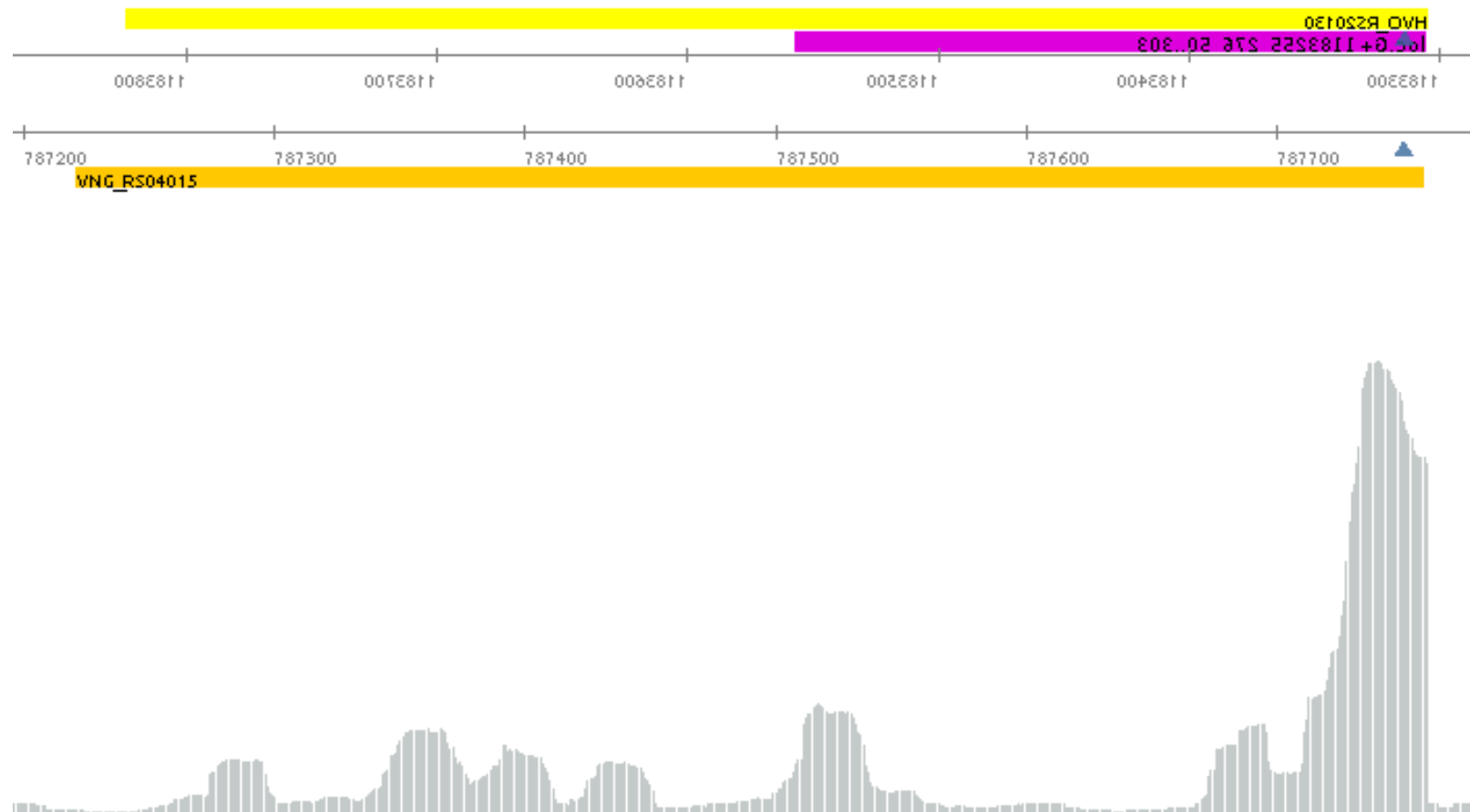

VNG\_RS04015

(c)

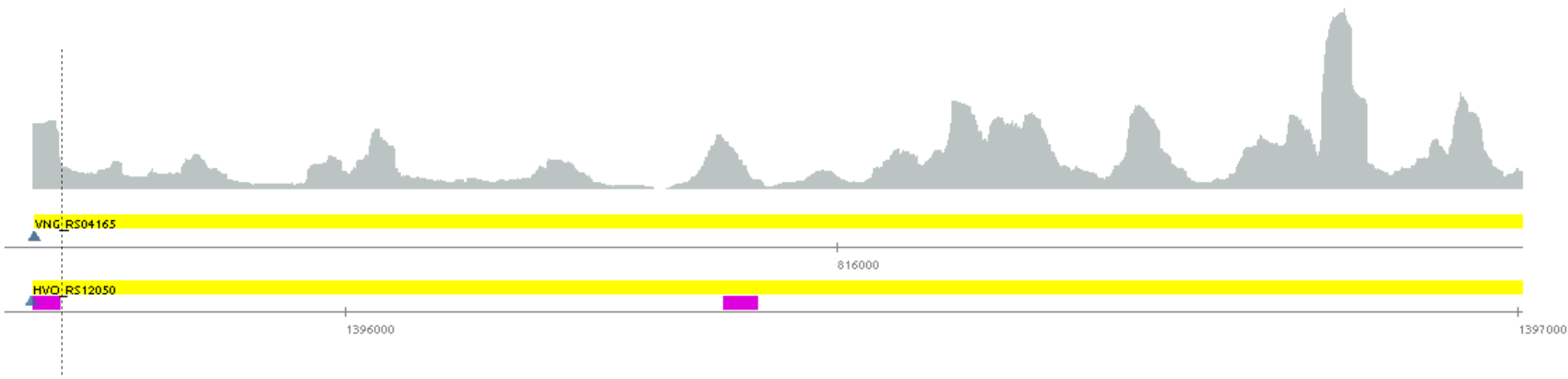

VNG\_RS04165

(d)

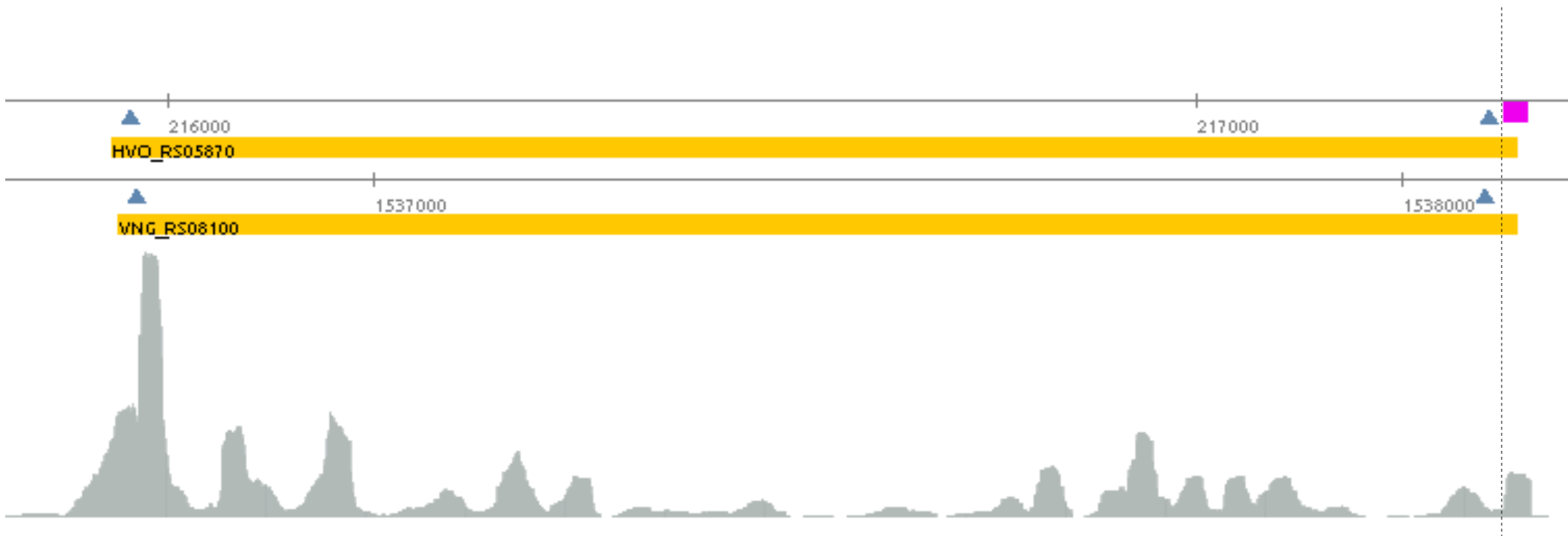

VNG\_RS08100

(e)

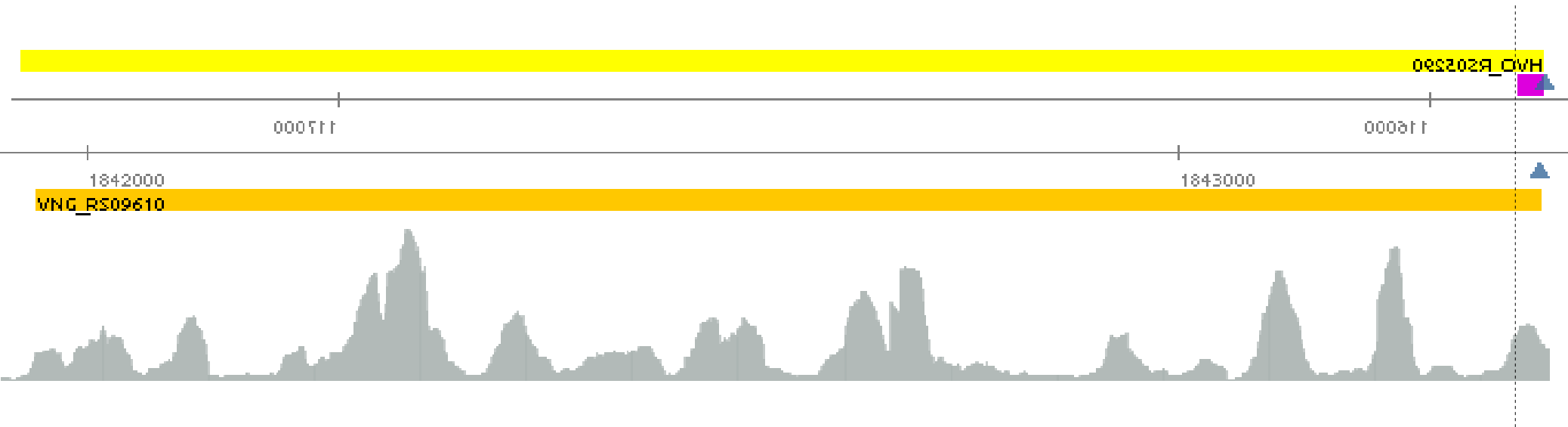

VNG\_RS09610

(f)

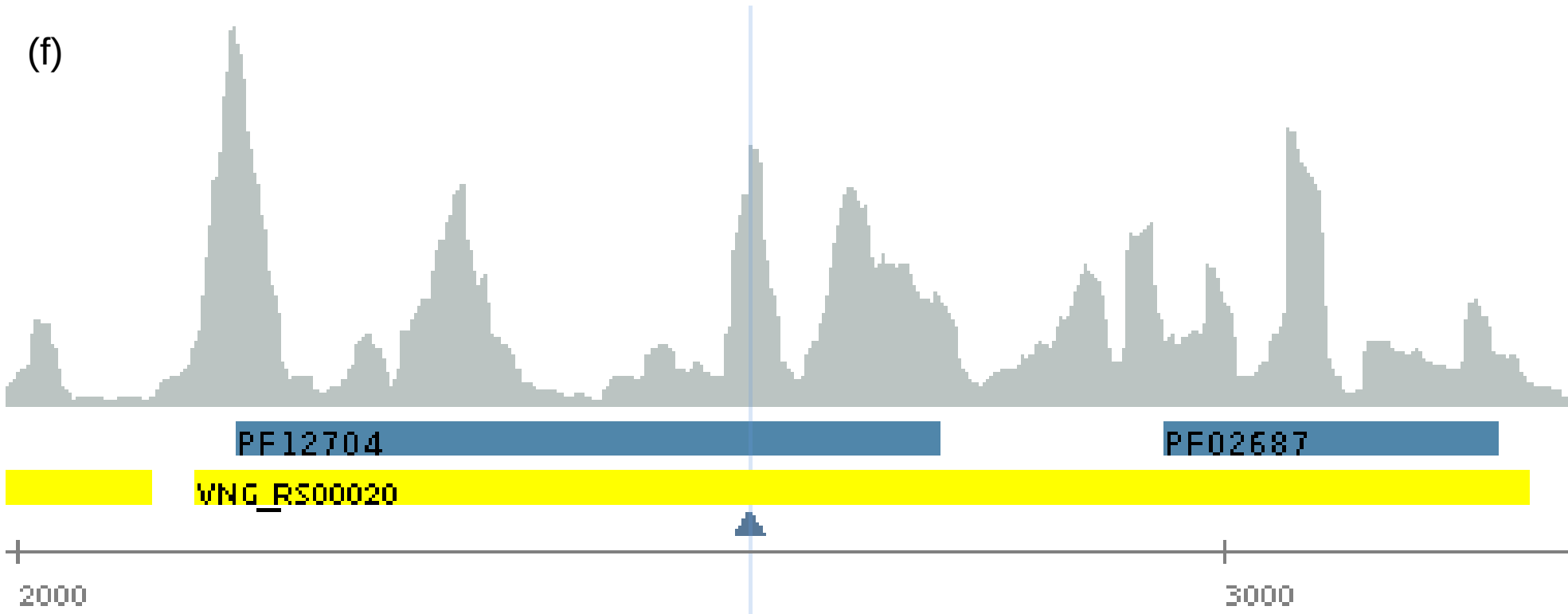

VNG\_RS00020

(g)

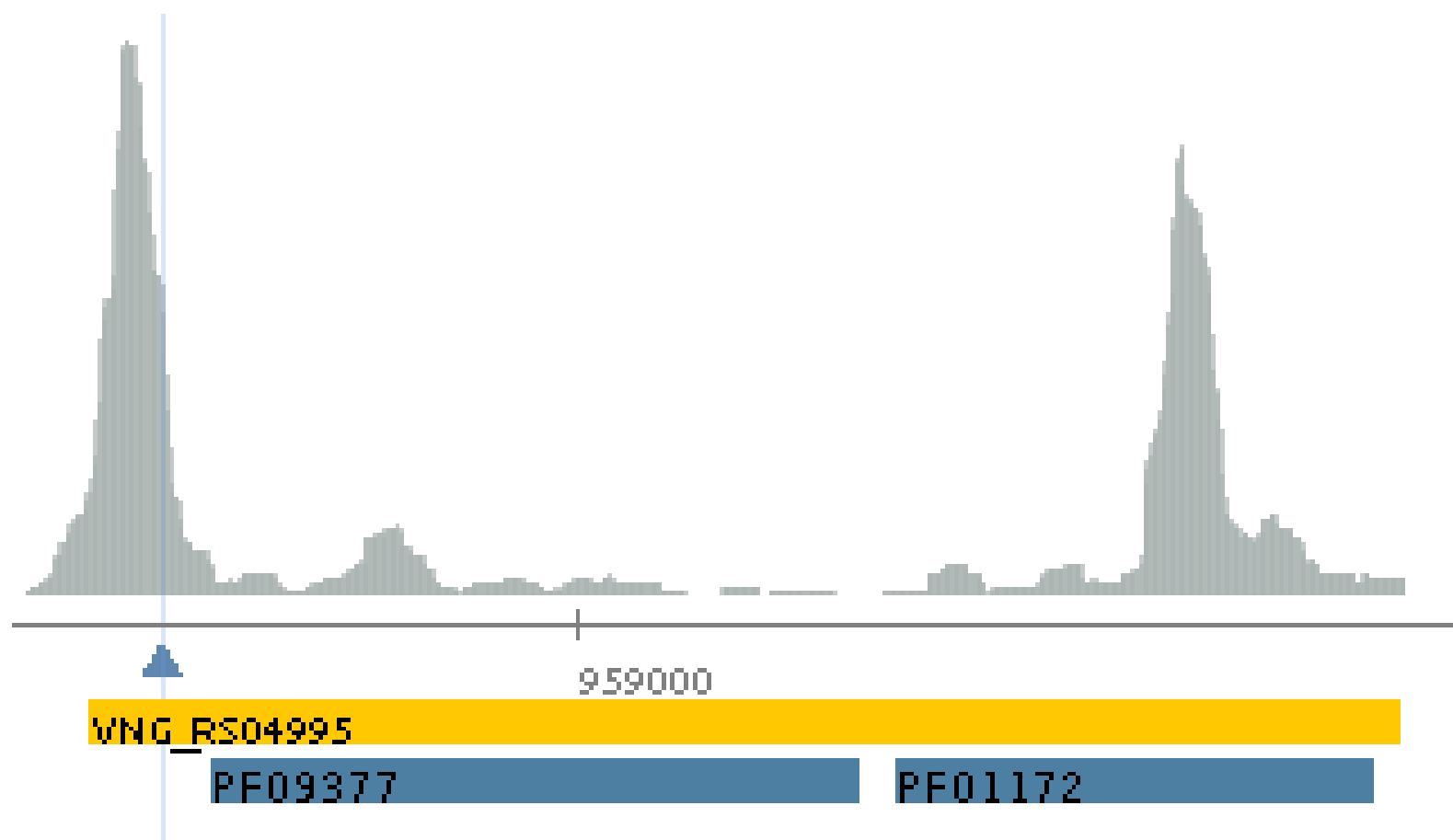

VNG\_RS04995

Supplement: Supplementary file 1 [file genes-12-01018-s001.zip › Amr_et_al_genes_2021_v39-zipfile/Figure_S7_v30.pdf]

# **Figure S6**

VNG\_RS11635

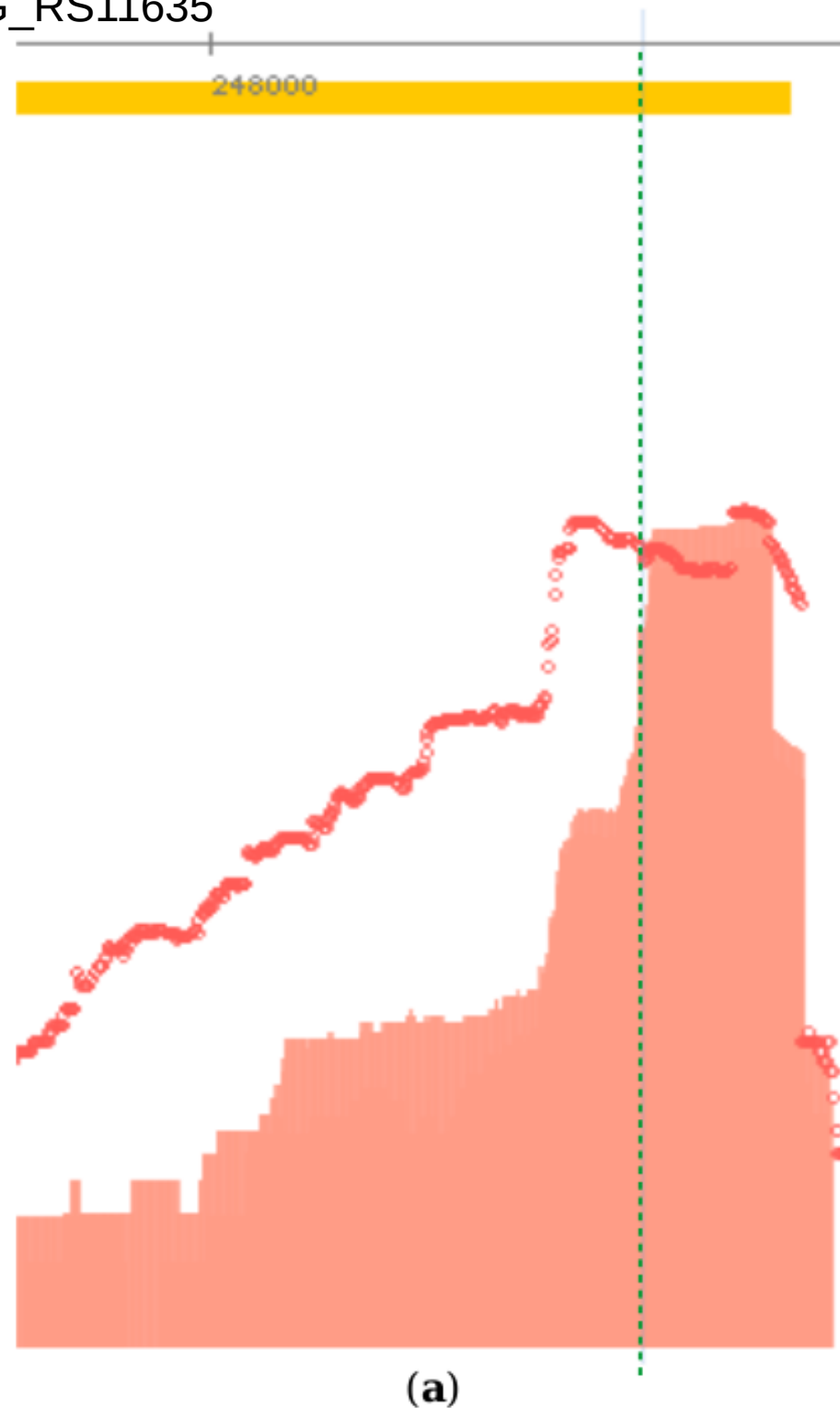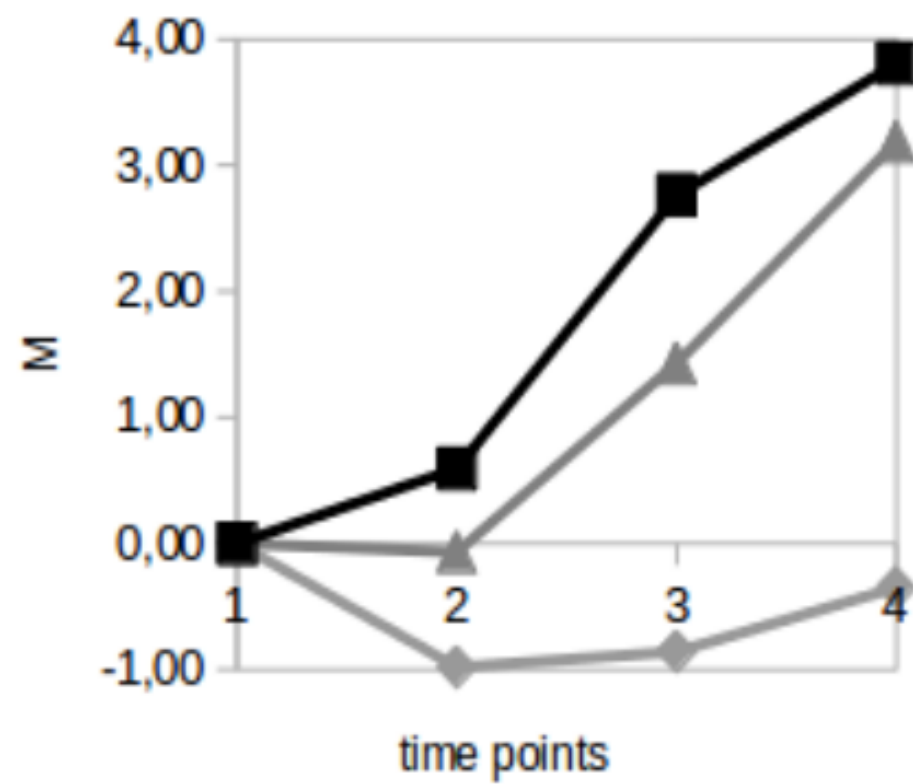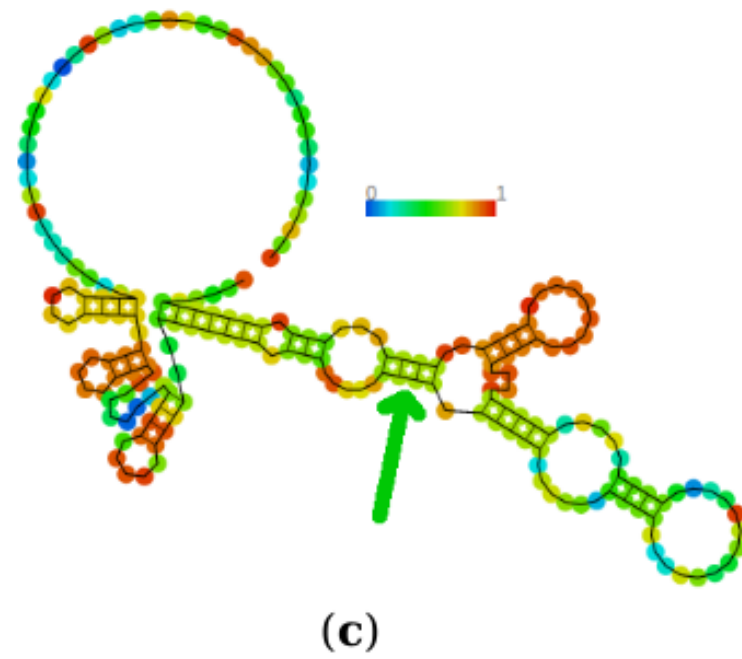

Supplement: Supplementary file 1 [file genes-12-01018-s001.zip › Amr_et_al_genes_2021_v39-zipfile/Figure_S6_v30.pdf]

# Figure S5

(a)

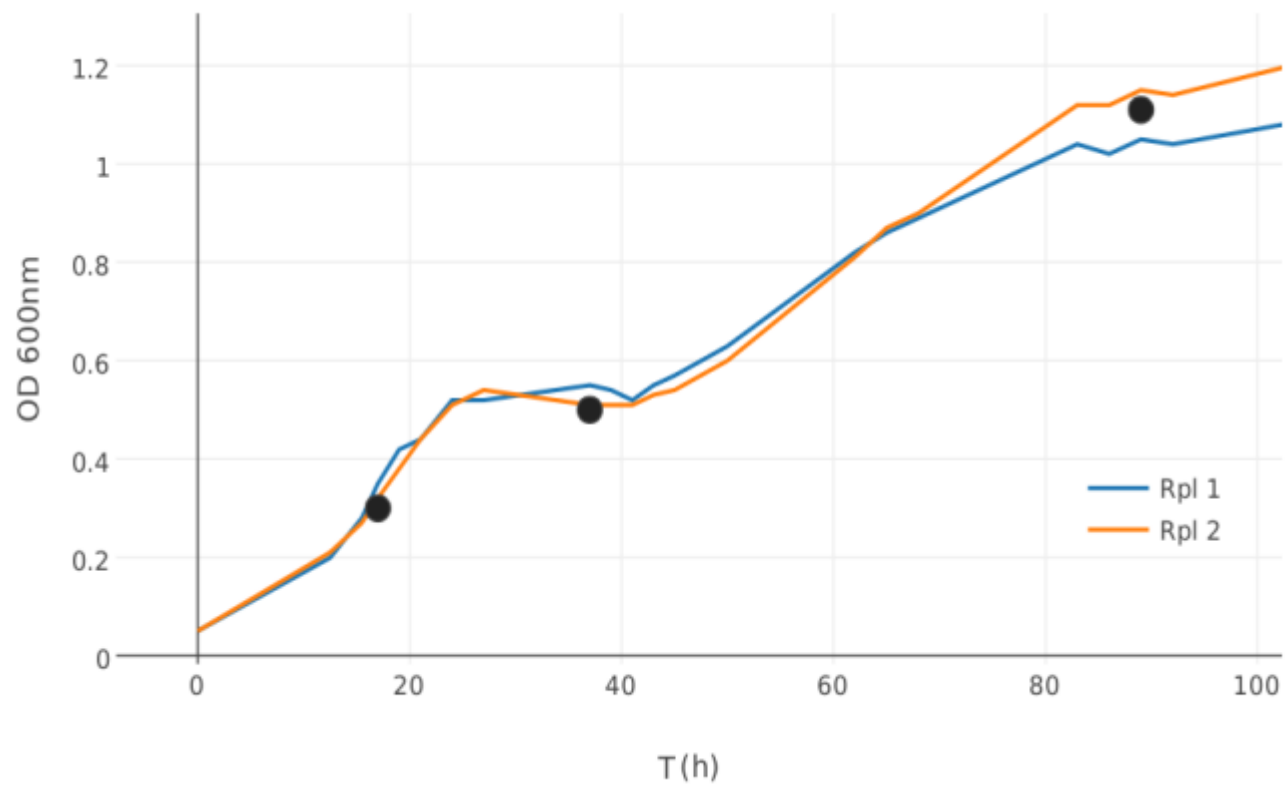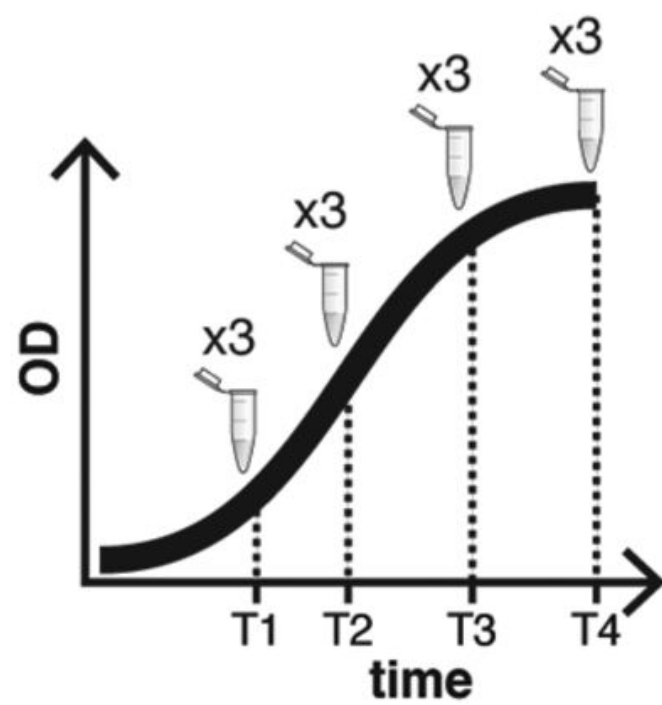

VNG\_RS10385

(b)

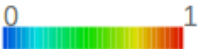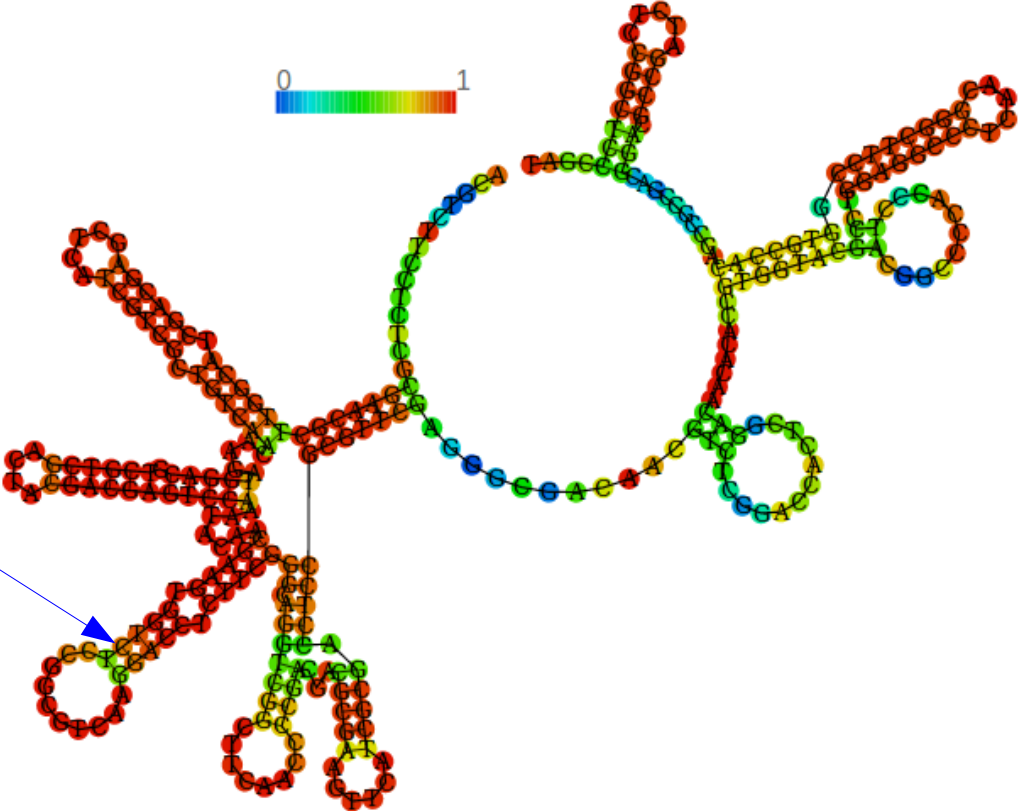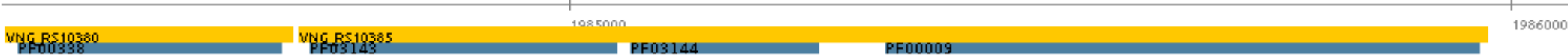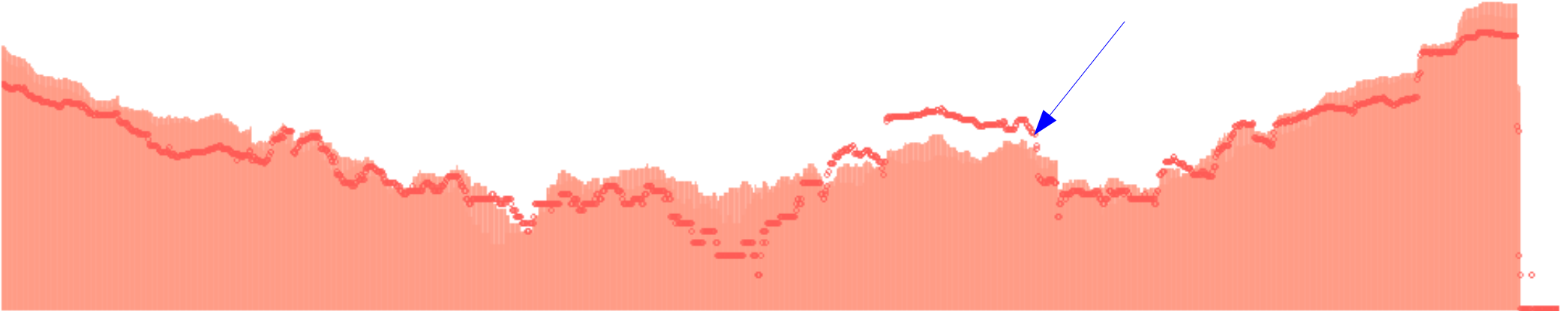

(c)

VNG\_RS03675

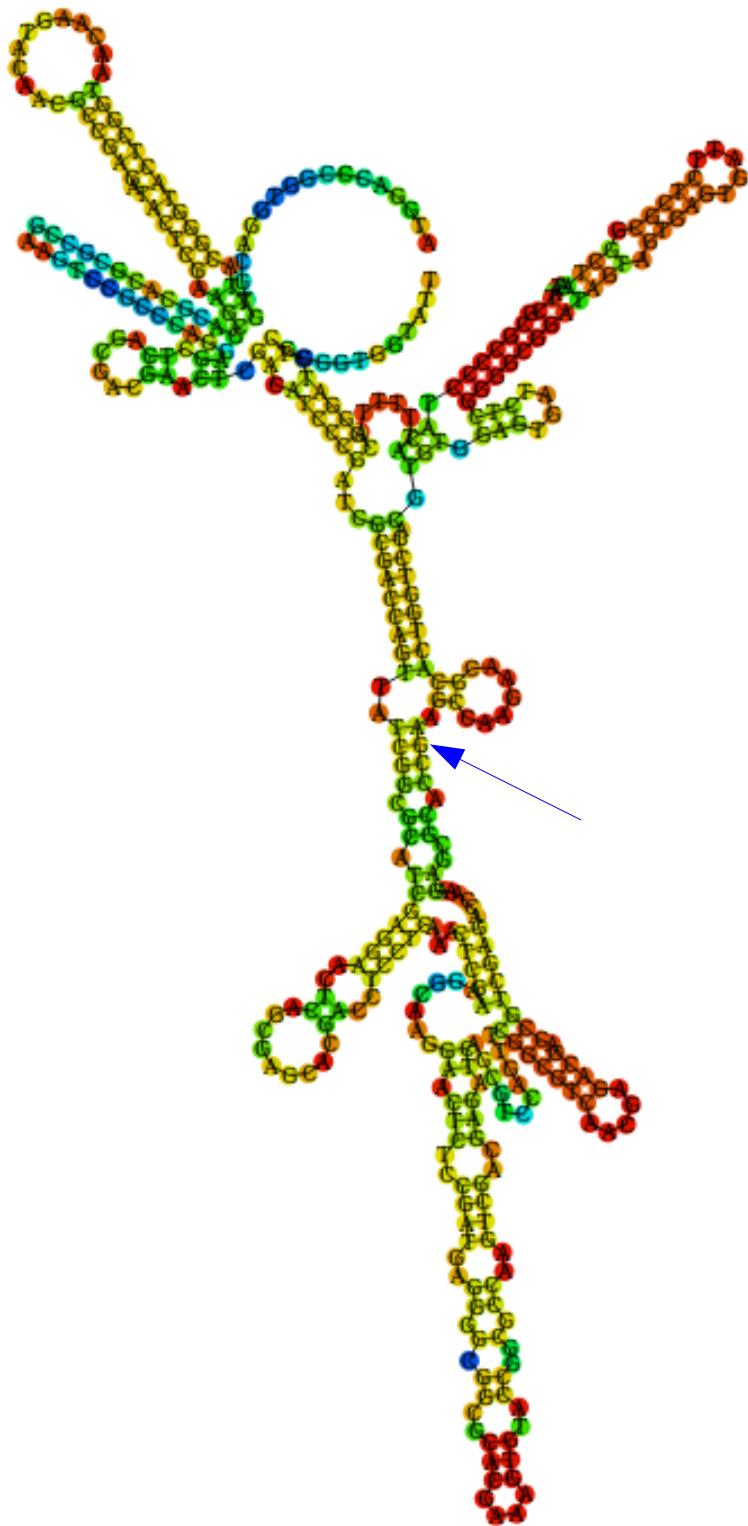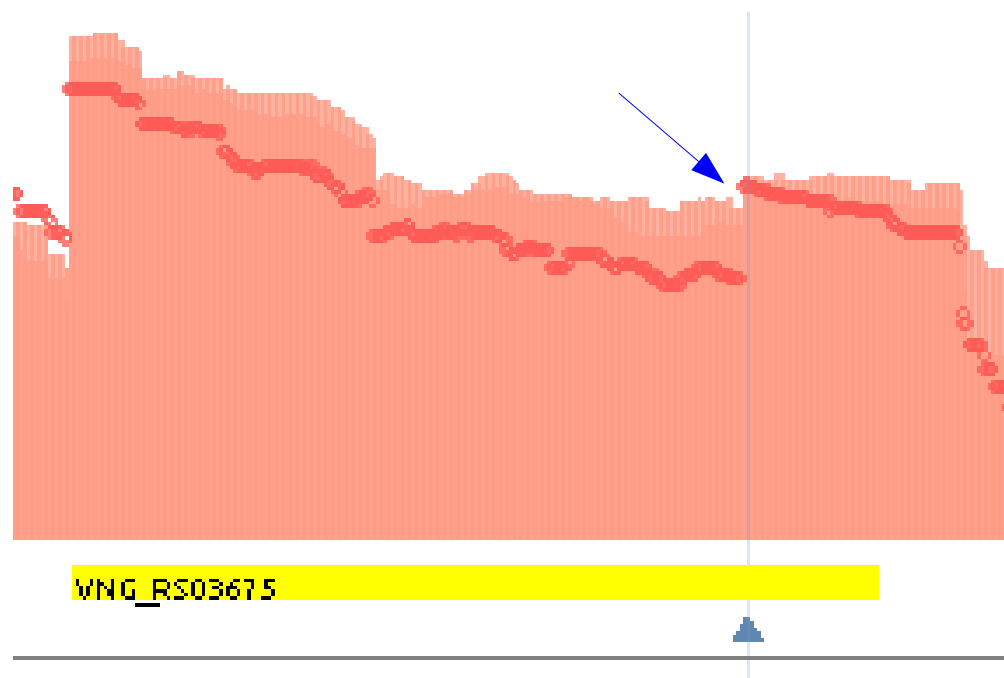

VNG\_RS08800

(d)

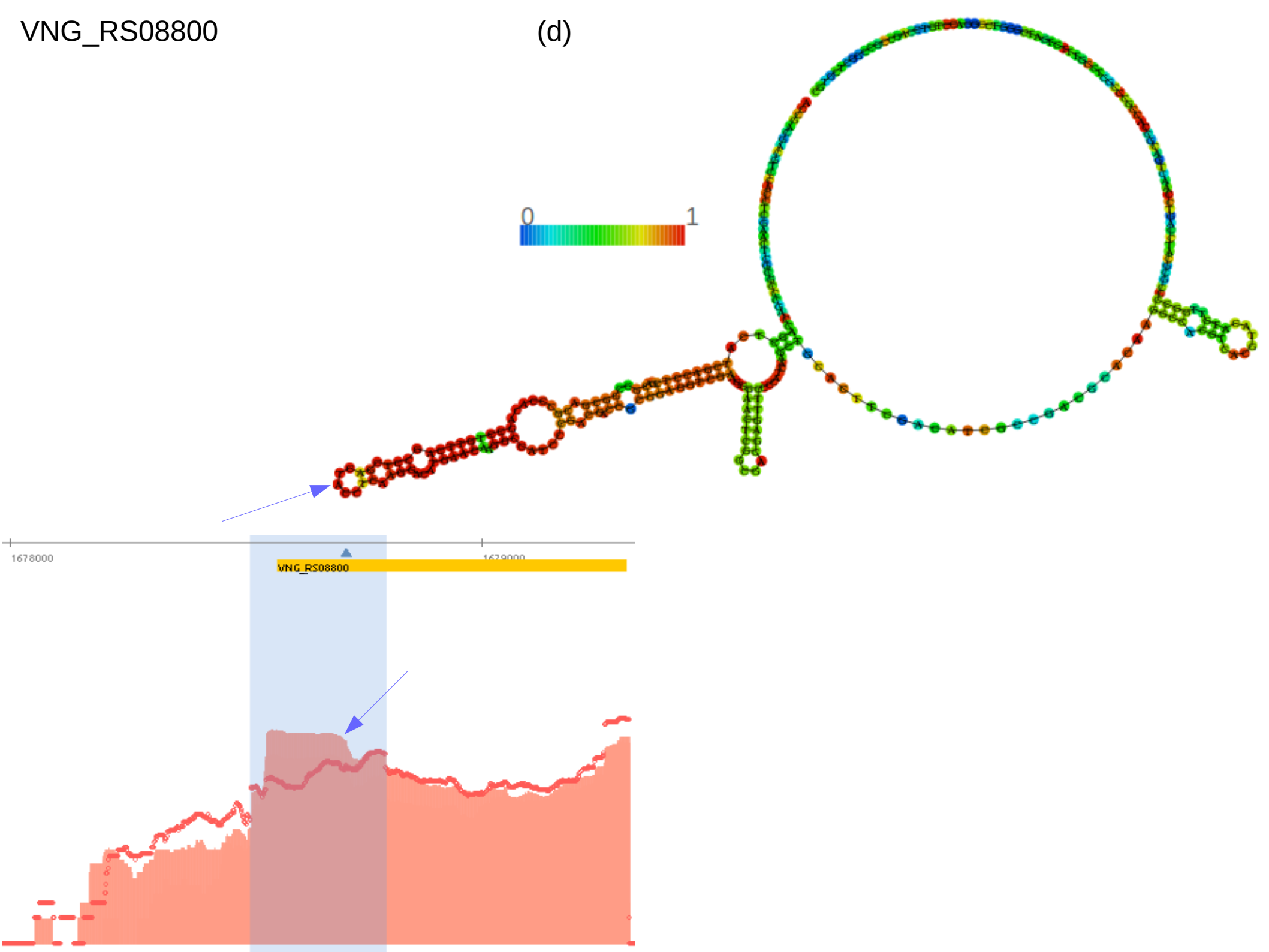

Supplement: Supplementary file 1 [file genes-12-01018-s001.zip › Amr_et_al_genes_2021_v39-zipfile/Figure_S5_v30.pdf]

# **Figure S3**

(a)

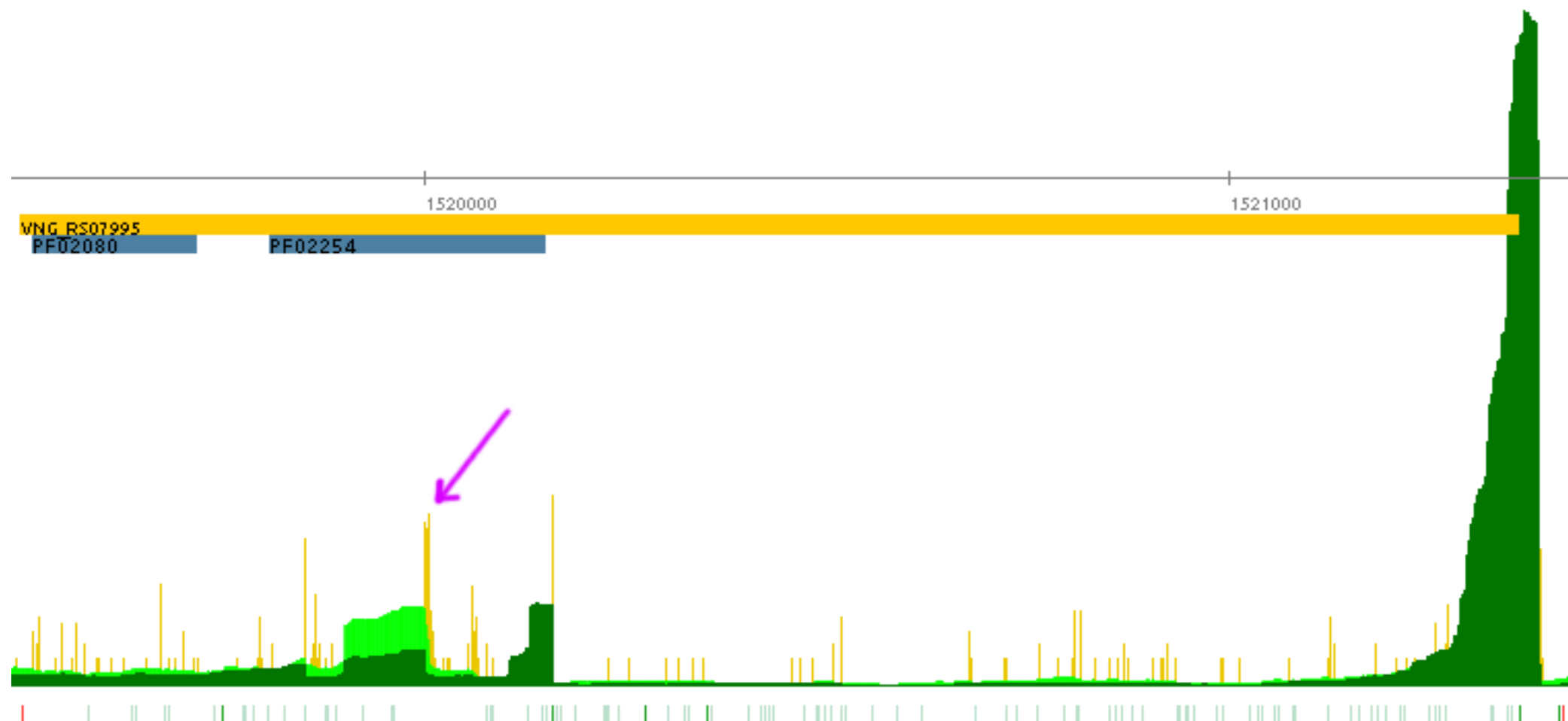

VNG\_RS07995

(b)

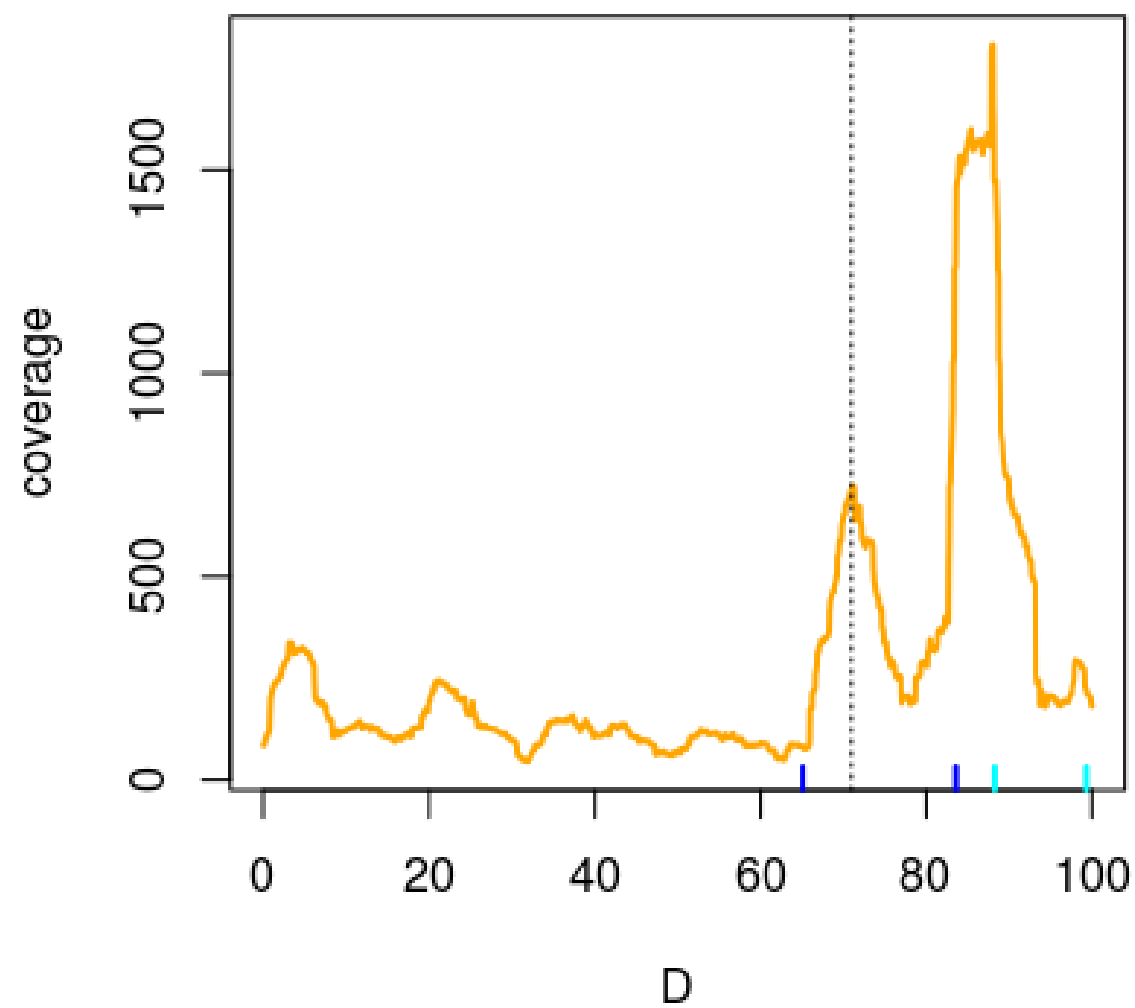

(c)

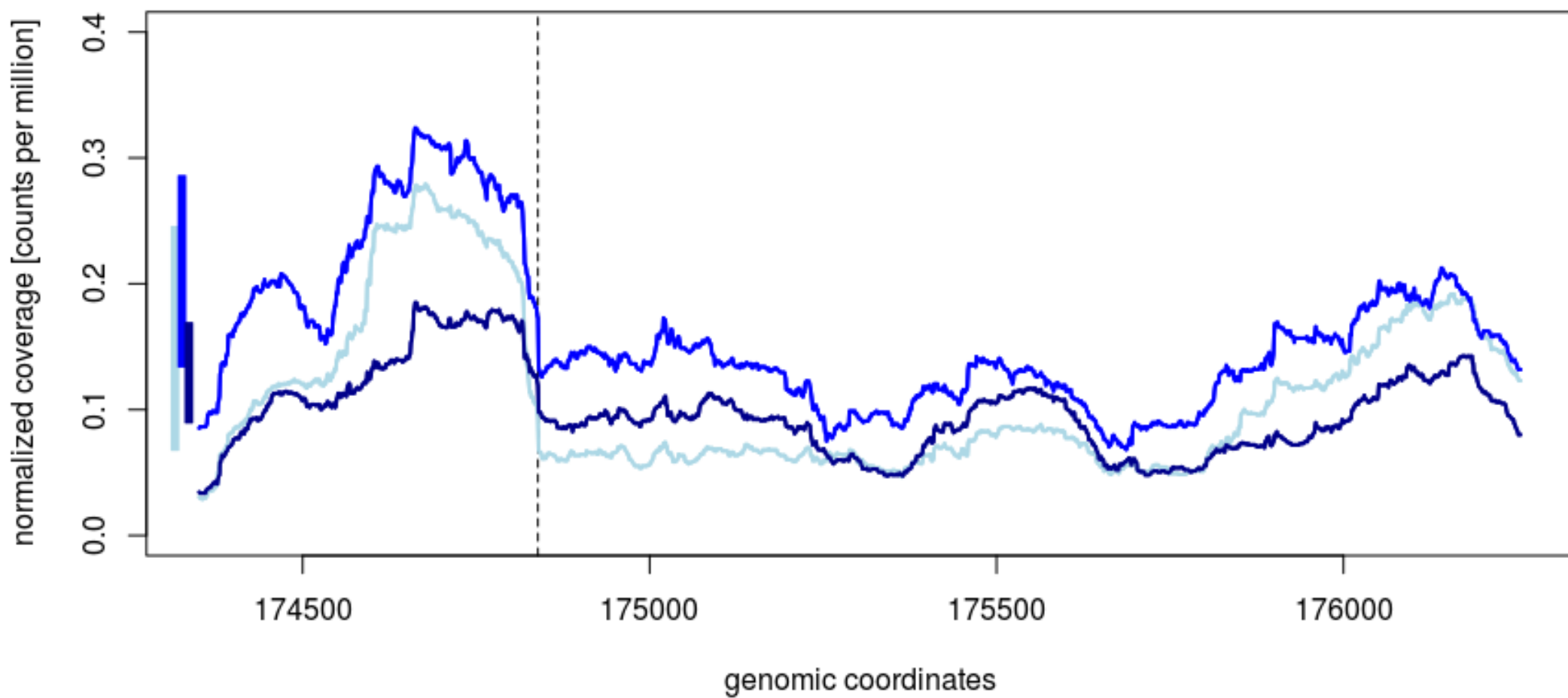

NJ7G\_RS00730

Supplement: Supplementary file 1 [file genes-12-01018-s001.zip › Amr_et_al_genes_2021_v39-zipfile/Figure_S3_v30.pdf]

# **Figure S2**

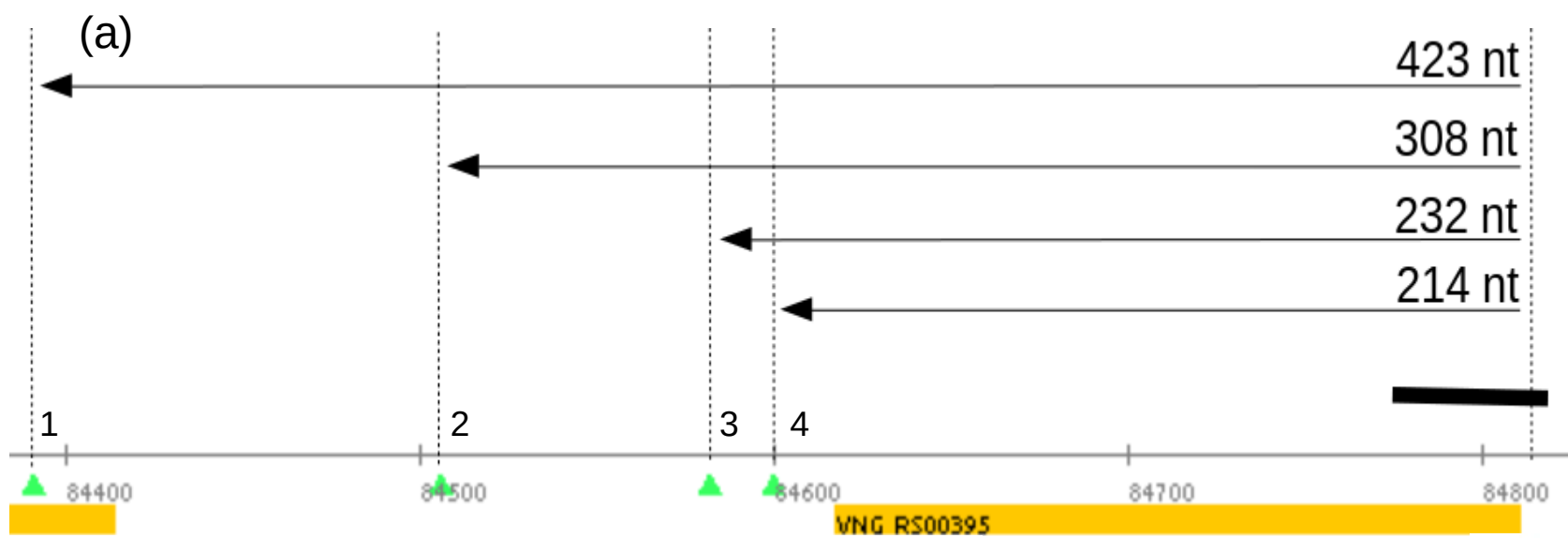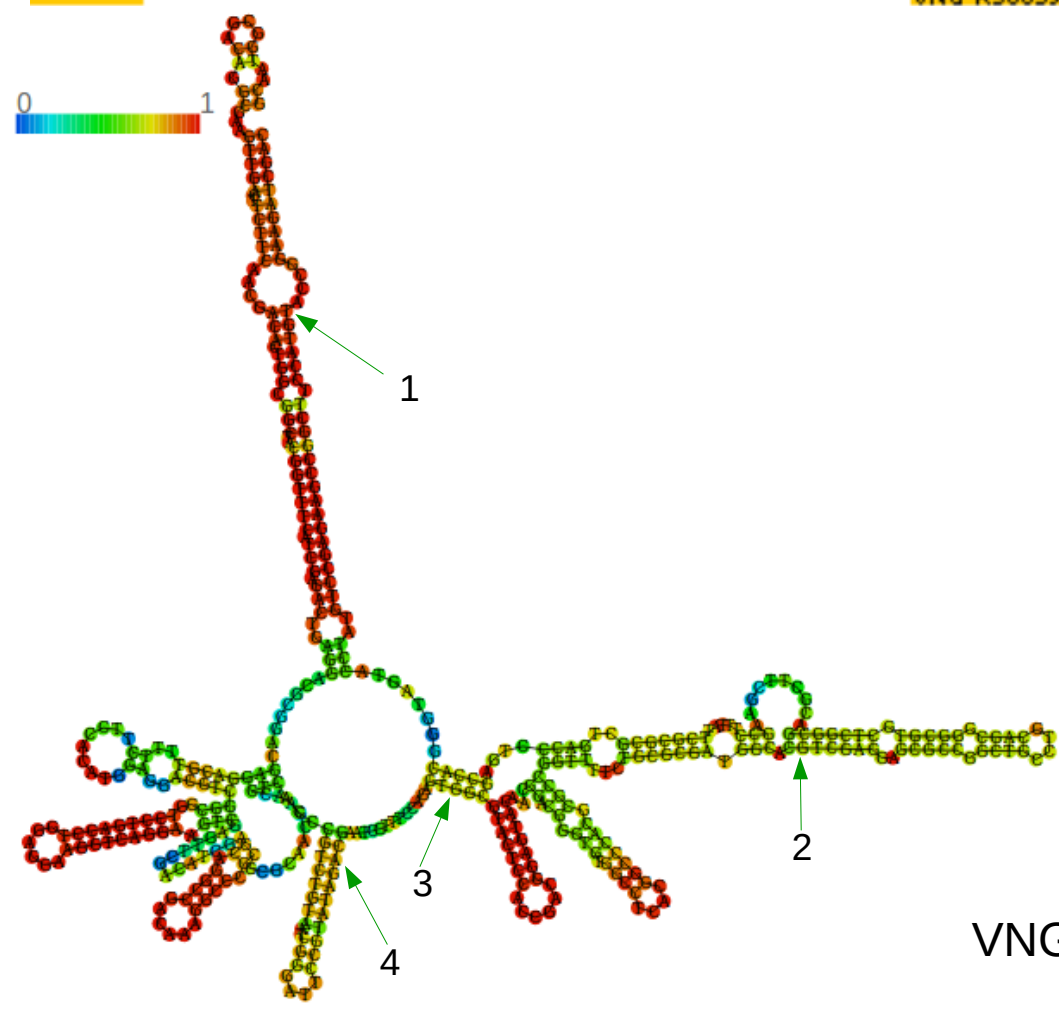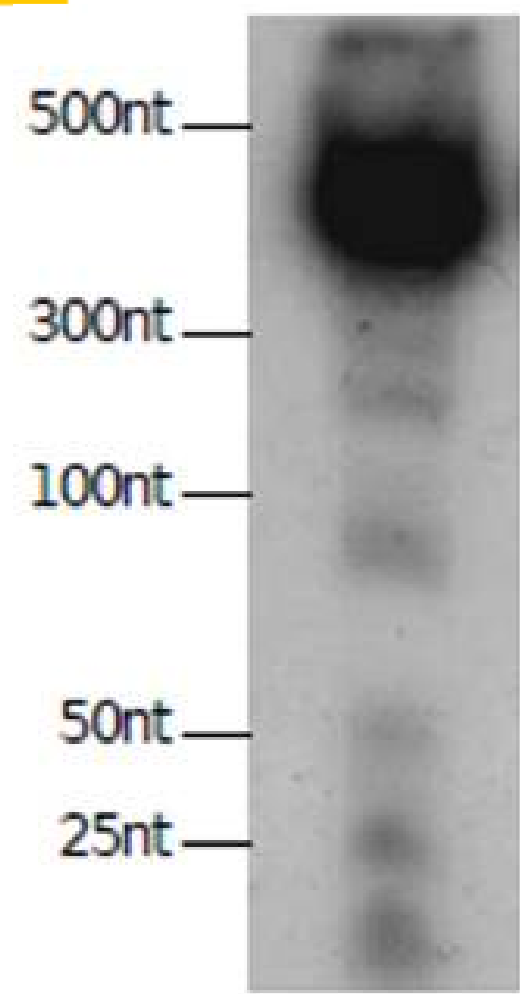

(b)

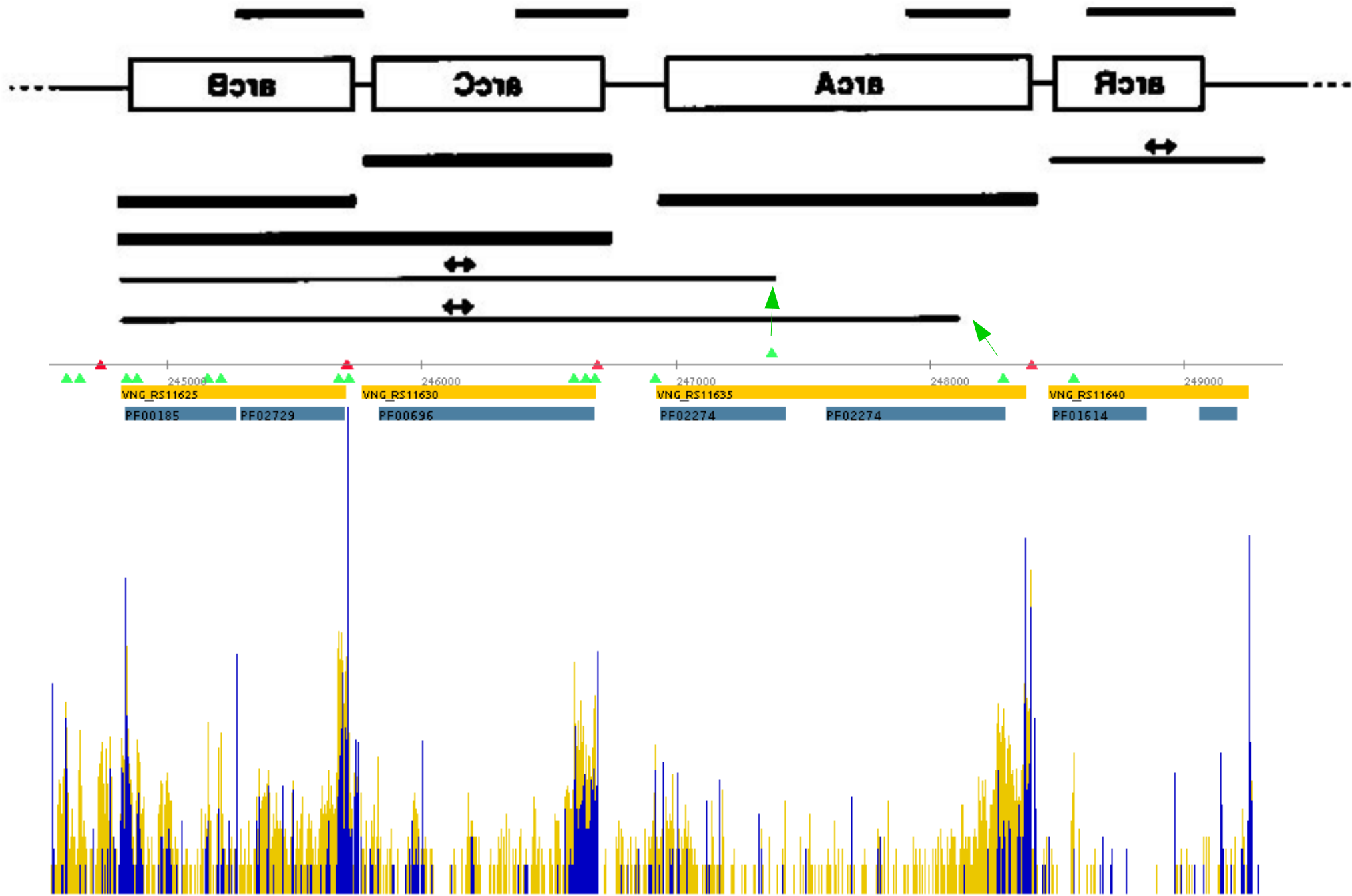

VNG\_RS11635

(c)

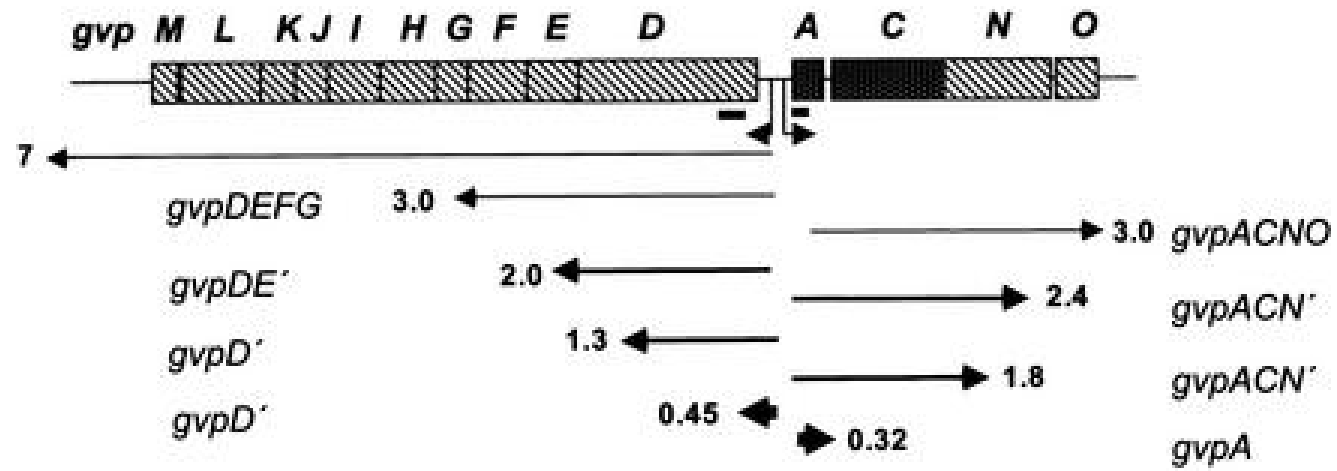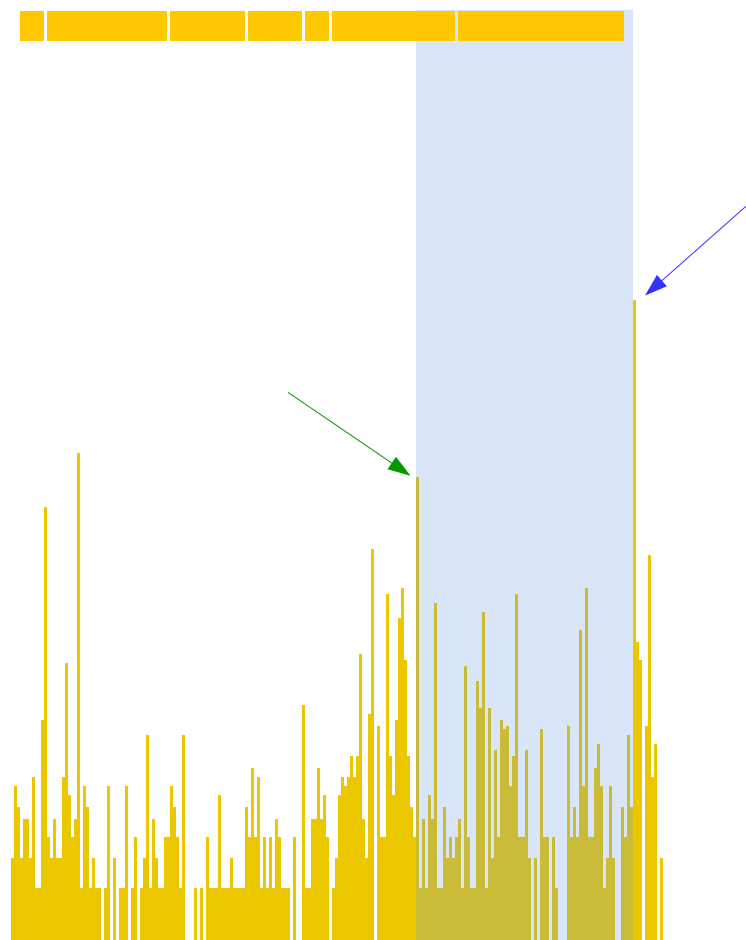

(d)

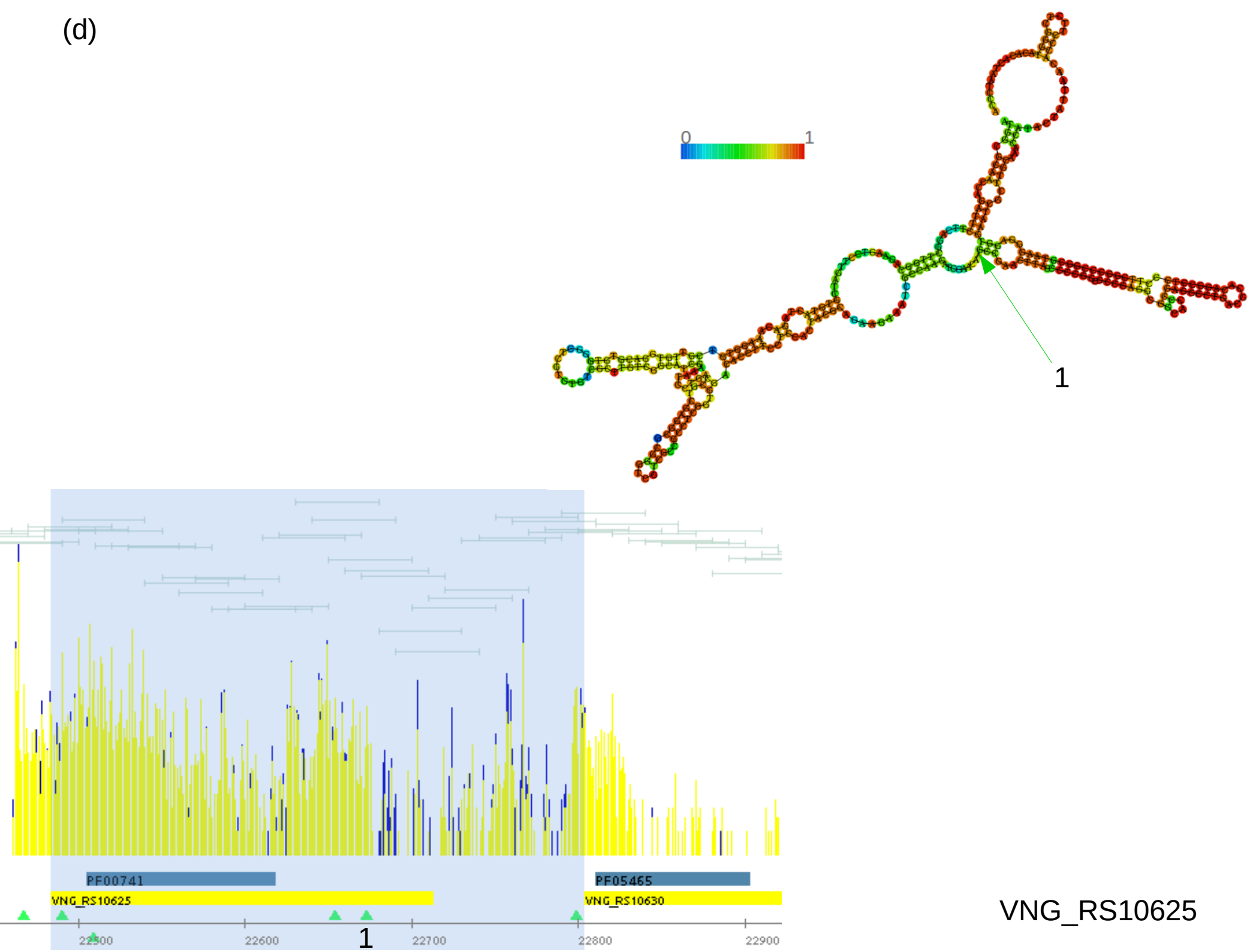

Supplement: Supplementary file 1 [file genes-12-01018-s001.zip › Amr_et_al_genes_2021_v39-zipfile/Figure_S2_v30.pdf]

# Figure S1

(a)

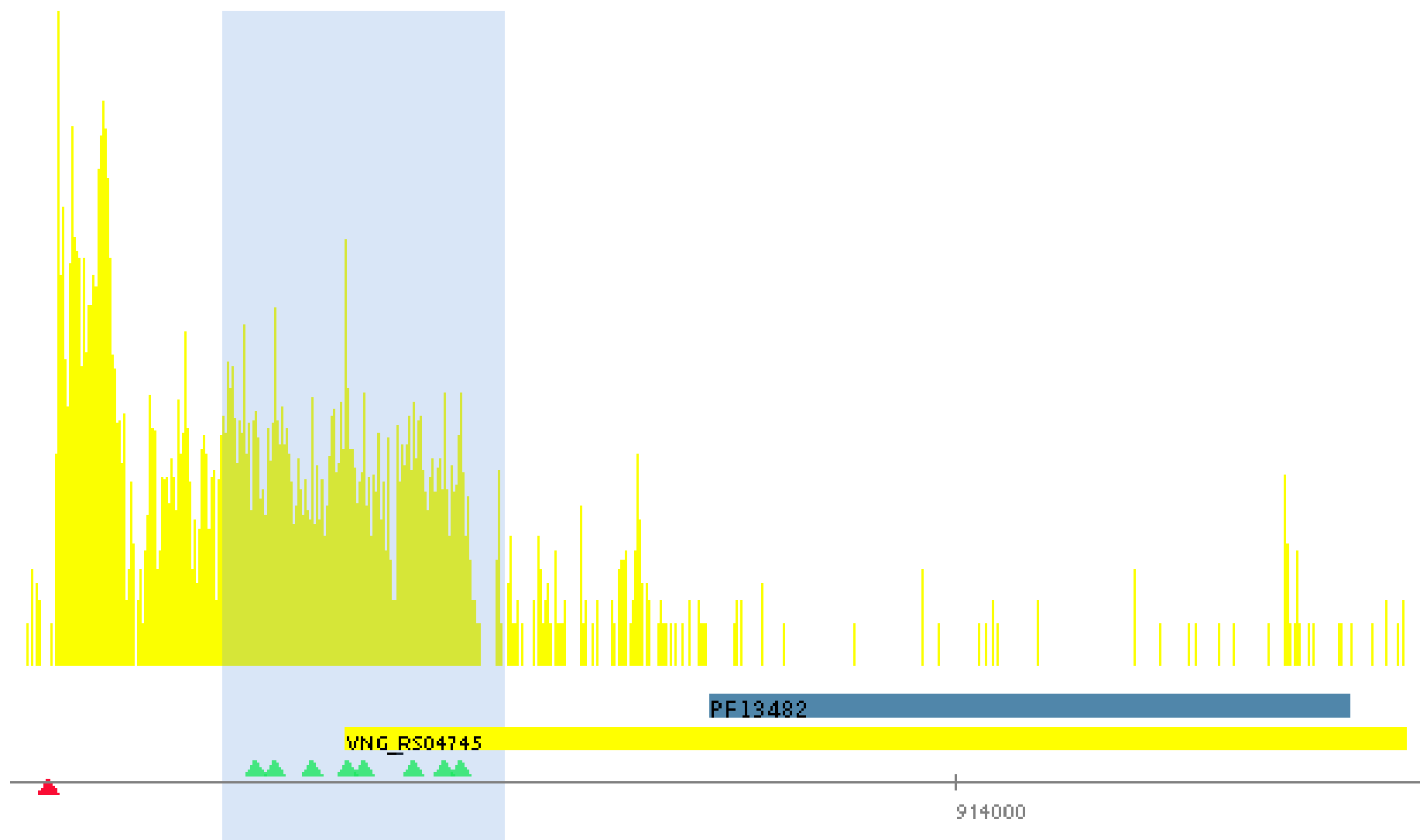

VNG\_RS04745

(b)

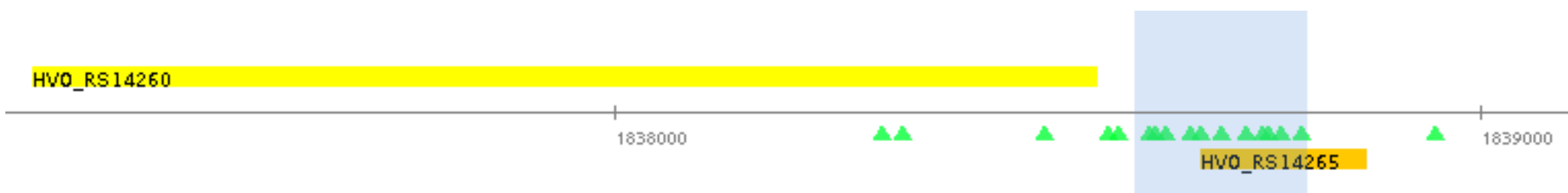

HVO\_RS14265

Supplement: Supplementary file 1 [file genes-12-01018-s001.zip › Amr_et_al_genes_2021_v39-zipfile/Figure_S1_v30.pdf]
